# Scientific evidence invalidates health assumptions underlying the FCC and ICNIRP exposure limit determinations for radiofrequency radiation: implications for 5G

**DOI:** 10.1186/s12940-022-00900-9

**Published:** 2022-10-18

**Authors:** International Commission on the Biological Effects of Electromagnetic Fields (ICBE-EMF), Igor Belyaev, Carl Blackman, Kent Chamberlin, Alvaro DeSalles, Suleyman Dasdag, Claudio Fernández, Lennart Hardell, Paul Héroux, Elizabeth Kelley, Kavindra Kesari, Don Maisch, Erica Mallery-Blythe, Ronald L. Melnick, Anthony Miller, Joel M. Moskowitz, Wenjun Sun, Igor Yakymenko

**Affiliations:** Tucson, USA

**Keywords:** Federal Communications Commission (FCC), International commission on non-ionizing radiation protection (ICNIRP), Radiofrequency radiation (RFR), Exposure limits, Exposure assessment, Radiation health effects, Reactive oxygen species (ROS), DNA damage, 5G, Scientific integrity, Cell phone*, Mobile phone*

## Abstract

In the late-1990s, the FCC and ICNIRP adopted radiofrequency radiation (RFR) exposure limits to protect the public and workers from adverse effects of RFR. These limits were based on results from behavioral studies conducted in the 1980s involving 40–60-minute exposures in 5 monkeys and 8 rats, and then applying arbitrary safety factors to an apparent threshold specific absorption rate (SAR) of 4 W/kg. The limits were also based on two major assumptions: any biological effects were due to excessive tissue heating and no effects would occur below the putative threshold SAR, as well as twelve assumptions that were not specified by either the FCC or ICNIRP. In this paper, we show how the past 25 years of extensive research on RFR demonstrates that the assumptions underlying the FCC’s and ICNIRP’s exposure limits are invalid and continue to present a public health harm. Adverse effects observed at exposures below the assumed threshold SAR include non-thermal induction of reactive oxygen species, DNA damage, cardiomyopathy, carcinogenicity, sperm damage, and neurological effects, including electromagnetic hypersensitivity. Also, multiple human studies have found statistically significant associations between RFR exposure and increased brain and thyroid cancer risk. Yet, in 2020, and in light of the body of evidence reviewed in this article, the FCC and ICNIRP reaffirmed the same limits that were established in the 1990s. Consequently, these exposure limits, which are based on false suppositions, do not adequately protect workers, children, hypersensitive individuals, and the general population from short-term or long-term RFR exposures. Thus, urgently needed are health protective exposure limits for humans and the environment. These limits must be based on scientific evidence rather than on erroneous assumptions, especially given the increasing worldwide exposures of people and the environment to RFR, including novel forms of radiation from 5G telecommunications for which there are no adequate health effects studies.

**Supplementary Information:**

The online version contains supplementary material available at 10.1186/s12940-022-00900-9.

## Introduction

In establishing exposure limits for toxic or carcinogenic agents, regulatory agencies generally set standards that take into account uncertainties of health risks for the general population [1] and for susceptible subgroups such as children [2]. That approach has not been applied in the same way to the setting of exposure limits for radiofrequency radiation (RFR) (frequency range: 3 kHz to 300 GHz). Moreover, assumptions underlying the current RFR exposure limits are flawed; hence, the limits that are currently applied do not adequately protect human and environmental health. This issue is discussed in greater detail under Assumption #9.

The Federal Communications Commission’s (FCC) limits for maximum permissible exposure to RF electromagnetic fields (EMF) [3] were established in 1996 [4], and currently include many recommendations from the International Commission on Non-Ionizing Radiation Protection [5]. These exposure limits were expected to protect against adverse health effects in humans that might occur from short-term (i.e., acute) exposures to RFR and have been maintained by the FCC for the past 26 years. The exposure limits that were established by the FCC in 1996 relied on criteria recommended by the National Council on Radiation Protection & Measurements (NCRP) [6] and the Institute of Electrical and Electronics Engineers (ANSI/IEEE) [7, 8]. The limits were “based on a determination that potentially harmful biological effects can occur at a SAR (specific absorption rate) level of 4.0 W/kg as averaged over the whole-body.” The SAR is a measure of the rate of RF energy absorbed per unit mass.

The threshold for a behavioral response and for acute thermal damage in sensitive tissues was considered to be an exposure that produced a whole-body SAR greater than 4 W/kg. In parallel with the development of the FCC’s RFR exposure limits, ICNIRP’s guidelines for limiting exposure to RF-EMF were also based on behavioral studies conducted in rats and monkeys in the 1980s [9].

The harmful effects that served as the basis for the exposure criteria were changes in behavior observed in small numbers of rats and monkeys when exposed to RFR for up to 60 minutes to power densities at which the whole-body SAR was approximately 4 W/kg or higher [10, 11]. Those studies were conducted in the early 1980s (1980 and 1984, respectively) by investigators of the US Navy Department. Consequently, 4 W/kg was identified as the threshold SAR for adverse health effects induced by RFR. In food-deprived monkeys that were exposed to three different frequencies (225 MHz, 1.3 GHz, and 5.8 GHz) during 60-min sessions, lever-pressing response rates for the delivery of food pellets were reduced compared to sham exposure sessions. The threshold SAR for this decreased response was reported to range from 3.2 to 8.4 W/kg [11]. Similarly, in food-deprived rats exposed to 40-min sessions at 1.28 or 5.62 GHz radiation, the threshold SAR for a decrease in response rate was reported to range from approximately 3.8 to 4.9 W/kg [10]. In experimental studies in which monkeys were exposed in an anechoic chamber for 4 hours to 1.29 GHz radiation at various power densities, an increase in mean body temperature of 0.7 °C was associated with a whole-body SAR of 4 W/kg [12]. Behavior disruption associated with an increase in body temperature of approximately 1.0 °C was assumed to be the most sensitive measure of harmful effects from RF-EMF exposure.

After establishing 4 W/kg as the threshold dose for acute harmful effects, both the FCC [3, 4] and ICNIRP [5, 9] set exposure limits for controlled occupational exposures to 0.4 W/kg SAR averaged over the whole body (based on applying a 10-fold safety/uncertainty factor). For the general population, the FCC’s and ICNIRP’s exposure limits were set at 0.08 W/kg SAR averaged over the whole body (by applying an additional 5-fold safety/uncertainty factor) for frequencies between 3 MHz and 3 GHz. The exposure limits established by the FCC and ICNIRP do not account for any impact of differing signal characteristics, such as carrier wave modulations or pulsing of the signal. Whole-body exposures for the general population are based simply on power levels averaged over 30-minute periods [3, 5].

Based on SAR distributions from whole-body exposures in which local (i.e., partial body) SARs were estimated to be 10 to 20 times the average value, local exposure limits were set 20 times higher than the average whole-body exposure limit [4–7]. For occupational exposures, local peak exposure limits were permitted up to 8 W/kg averaged over any 1-g cube of tissue [4] or 10 W/kg averaged over any 10 g of contiguous tissue [9] by the FCC and ICNIRP, respectively. For the general population, local peak SARs for partial-body exposures were not to exceed 1.6 W/kg averaged over any 1 g of cube-shaped tissue [3], or not to exceed 2.0 W/kg averaged over any 10 g of cube-shaped tissue [5]. Higher limit values are permissible for extremities. Extremities include the hands, wrists, feet, ankles, and pinnae (the external part of the ear), despite the close proximity of the ear to the brain. These adjustments were made long before the widespread use of wireless communication devices in which the emitting antenna is typically held close to local body organs such as the brain. The NCRP document [6] acknowledges that exposures could be greater than the recommended safety limit values when people are in close proximity to emitters of RFR.

The setting of exposure limits for the prevention of excessive tissue heating was based on the following assumptions: 1) electromagnetic waves at frequencies used in wireless communications do not have sufficient energy to break chemical bonds or ionize molecules [13]; 2) RFR could not damage DNA; and 3) tissue heating was the only possible biological effect of nonionizing radiation [5, 9, 14–16]. For potential environmental and human health issues that are not addressed in the setting of exposure limits (for example effects of chronic exposures, or effects of co-exposure of skin to RFR and other environmental agents, such as would occur with 5G exposure in combination with sunlight), the implicit assumption is that such effects do not matter, or that the arbitrarily selected safety/uncertainty factor is sufficient to deal with those concerns. In any case, it is expected that underlying assumptions applied to health risk assessments would be clearly described [1].

Exposure limits for RF radiation are based on numerous assumptions; however, research studies published over the past 25 years show that most of those assumptions are not supported by scientific evidence. In the NCRP report [6], the authors noted that when further understanding of biological effects of RF radiation becomes available, exposure guidelines will need to be evaluated and possibly revised. The ANSI/IEEE document [7] also notes that effects of chronic exposure or evidence of non-thermal interactions could result in revising exposure standards. Unfortunately, these recommendations were never implemented. Assumptions of safety from exposures that could adversely affect human or environmental health should be tested and validated *before* widespread exposures occur, not afterwards, by agencies responsible for protecting public health.

**Fig. 1:**
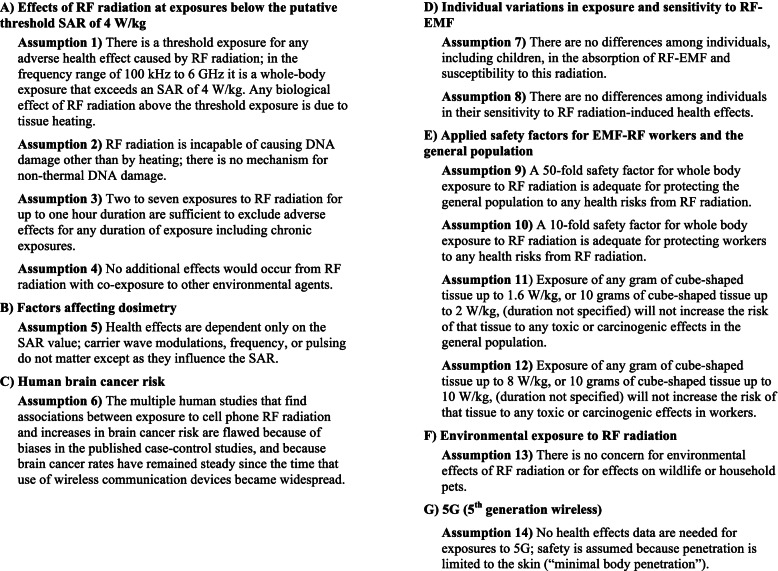
Assumptions Underlying the FCC/ICNIRP Exposure Limits for RF Radiation

In this paper, we highlight studies that demonstrate the fallacy of inherent assumptions in the FCC/ICNIRP guidelines for RF radiation exposure limits, and we find that the limits fail to protect human and environmental health. Fourteen assumptions that underlie the RFR exposure limits established in the 1990s and reaffirmed in 2020 by the FCC [4, 5] and ICNIRP [5, 9] are addressed in this paper and are shown in Fig. 1.

## Assumptions underlying exposure limits for RF radiation and the scientific evidence demonstrating that these assumptions are not valid

### A. Effects of RF radiation at exposures below the putative threshold SAR of 4 W/kg

- **Assumption 1**) *There is a threshold exposure for any adverse health effect caused by RF radiation; in the frequency range of 100 kHz to 6 GHz it is a whole-body exposure that exceeds an SAR of 4 W/kg. Any biological effect of RF radiation above the threshold exposure is due to tissue heating.*

#### Cardiomyopathy and carcinogenicity

In response to a request from the Food and Drug Administration’s (FDA) Center for Devices and Radiological Health [17], the National Toxicology Program (NTP) conducted toxicity and carcinogenicity studies of cell phone (CDMA- or GSM-modulated) radiation in rats and mice exposed to RFR at frequencies of 900 MHz and 1800 MHz, respectively [18, 19]. Exposures to RFR for up to 2 years occurred in reverberation chambers over 18 hours/day on a continuous cycle of 10 minutes on and 10 minutes off. In rats, the whole-body SAR levels during the 10-minute on cycles were 0, 1.5, 3, or 6 W/kg.

The major histopathological findings from the NTP study in male rats [18] included dose-related increases in cardiomyopathy, increased incidence of cancers and preneoplastic lesions in the heart (schwannoma and Schwann cell hyperplasia) and brain (glioma and glial cell hyperplasia), increases in prostate gland tumors and hyperplasias, significant increases in adrenal gland tumors, and significant increases in the overall incidence of benign or malignant neoplasms in all organs in the 3 W/kg groups. The incidence of cardiomyopathy was also increased in GSM-exposed female rats, and significant increases in DNA damage were found in rats and mice [18, 19]. Similarly, an earlier study by Chou et al. [20] found a significant (3.6-fold) increase in the incidence of primary malignant neoplasms in male rats exposed to 2450 MHz pulsed RFR for 25 months (21.5 hr./day) at an SAR that ranged from 0.15 to 0.4 W/kg.

A 3-day external peer-review of the NTP studies confirmed there was “clear evidence of carcinogenic activity” in male rats for heart schwannomas, and “some evidence of carcinogenic activity” for brain gliomas and adrenal gland tumors with exposure to either GSM- or CDMA-modulated RF radiation [21]. In addition, a lifetime study by the Ramazzini Institute reported a significant increase in heart schwannomas in male rats exposed 19 hour/day to 1800 MHz GSM-modulated RFR at a field strength of 50 V/m, equivalent to a whole-body SAR of 0.1 W/kg [22]. The incidence of heart Schwann cell hyperplasia was also increased in that exposure group. These findings are consistent with results from the NTP study and demonstrate that the proliferative effect of modulated RFR in heart Schwann cells is a reproducible finding that can occur at doses far below the assumed whole-body threshold SAR of 4 W/kg.

ICNIRP [23] dismissed the evidence of carcinogenicity for RFR that was provided in the studies by the NTP [18] and the Ramazzini Institute [22] based on their earlier critique of those studies [24]. However, that critique demonstrated an unfortunate lack of understanding together with a misrepresentation of the design, conduct, and interpretation of experimental carcinogenicity studies in animal models [25], as well as a lack of appreciation for the remarkable concordance between the tumor responses observed in experimental animals with those identified in cancer epidemiology studies of mobile phone users described under Assumption #6.

Neither heating effects nor thermal stress was likely causal of the adverse health effects observed in the NTP [18] study, since there was no tissue damage observed in a 28-day study at the same SARs, there was no significant effect on body weight during the 2-year study, and there were no exposure-related clinical observations that would indicate thermal or metabolic stress. Furthermore, a preliminary thermal pilot study demonstrated that body temperatures did not increase by more than 1^O^ C at the exposure levels used in the chronic studies [26], and there is no evidence that a small change in body temperature associated with the RFR exposures in the NTP study can cause the types of carcinogenic effects that were observed. The similar findings of GSM-modulated RFR on Schwann cells by the Ramazzini Institute [22] at much lower whole-body SARs confirm these effects to be independent of tissue heating.

#### Neurological effects

Though the FCC and ICNIRP exposure limits are based on a putative threshold dose of 4 W/kg due to behavioral disruption observed at higher doses in rats and monkeys [10, 11] numerous studies have shown consistent and reproducible deficits in spatial learning and memory in laboratory animals exposed to RF radiation at SARs below 4 W/kg. Examples of study exposures that demonstrated these neurological effects included 900 MHz GSM at 0.41–0.98 W/kg, 2 hr./day for 4 days in mice [27]; 900 MHz GSM at 0.52–1.08 W/kg, 2 hr./day for 1 month in rats [28]; 900 MHz GSM at 1.15 W/kg, 1 hr./day for 28 days in rats [29]; 900 MHz pulsed RFR at 0.3–0.9 W/kg for 6 hr./day in rats from conception to birth and tested at 30 days of age [30]; 900 MHz GSM and 1966 MHz UMTS at 0.4 W/kg for 6 months in rats [31]; and 900 MHz continuous wave EMF at 0.016 W/kg 3 hr./day for 28 days in rats [32]. The studies cited above are not the only studies showing these effects, but they clearly demonstrate that exposure to RFR at an SAR of 4 W/kg is not a threshold dose for neurological effects in rodents. The effects of RF radiation on spatial learning and memory indicate the hippocampus as a target site of these exposures. For a more complete listing of neurological effects of RFR reported between 2007 and 2017 see Lai [33].

In addition, many studies have reported changes in brain electrical activities in human subjects, measured by electroencephalography (EEG), including sleep disturbance from single exposures to cell phone RF radiation. This is not surprising since the nervous system transmits messages based on electrical signals generated by nerve cells. Decreased β-trace protein, which is a key enzyme in the synthesis of a sleep-promoting neurohormone, has been seen in young adults with high-cumulative amounts of hours of mobile phone use [34]. Another frequently reported effect of RF radiation is increased blood-brain barrier permeability in rats at SARs much lower than 4 W/kg, e.g. [32, 35–41]. Oxidative stress induced in the brain of animals exposed to RF-EMF has been associated with observed neurological effects [42]. Although many studies did not observe significant changes in neurological effects in humans and several studies did not observe increased permeability in the blood-brain barrier in animal models [33], differences in EMF frequency, modulation, duration of exposure, and direction of incident waves to the exposed subject, as well as difference in dielectric properties and the size and shape of the exposed subject likely account for differences in observed effects [43, 44].

#### Sperm damage

The effect of non-ionizing microwave radiation on the testis (testicular degeneration in mice) was first reported 60 years ago [45]. Since then, and with the rapid increase in use of RF-EMF emitting devices, numerous studies have investigated testicular effects of RFR and potential associations with male infertility [46–50]. Human and animal studies have shown that the testis is one of the most sensitive organs to RF-EMF exposures, and that keeping a mobile phone in trouser pockets in talk mode can affect fertility parameters e.g., sperm motility, sperm count, sperm morphology, and apoptosis [48, 51]. Meta-analyses of published epidemiologic studies on the impact of mobile phone radiation on sperm quality in adult men have found significant decreases in sperm motility, sperm viability and/or sperm concentrations that were associated with mobile phone usage [52–55]. Several physical factors associated with exposure conditions can affect the outcome of human studies, including depth of energy penetration, duration of call, type of transmission technology, distance of the device to the body or testis, and power density with defined SAR. For example, Zilberlicht et al. [56] observed higher rates of abnormal sperm concentrations among men who held their phones less than 50 cm from their groin.

The effects of RFR on reproductive parameters in humans are consistent with results from experimental studies in animals and in vitro studies. For example, exposure of human semen to 850 MHz radiation from mobile phones for 1 hour at an SAR of 1.46 W/kg caused a significant decrease in sperm viability that was associated with an increase in reactive oxygen species (ROS) [50] or an increase in sperm DNA fragmentation [57]. Exposure of isolated human spermatozoa to 1.8 GHz RF-EMF significantly reduced sperm motility and induced ROS generation at an SAR of 1.0 W/kg, and significantly increased oxidative DNA damage and DNA fragmentation at an SAR of 2.8 W/kg [58].

Some examples of effects of RFR on male fertility factors in studies with experimental animals at SARs below 4 W/kg include: a decrease in sperm count and an increase in ROS in rats exposed to mobile phone frequencies 2 hr./day, for 35 days (SAR = 0.9 W/kg) [59]; increases in oxidative stress, 8-hydroxy-deoxyguanosine (8-OHdG), and DNA strand breaks in the testes of rats exposed to 900 MHz (SAR = 0.166 W/kg), 1800 MHz (0.166 W/kg), or 2100 MHz (0.174 W/kg) 2 hr./day for 6 months [60]; an increase in ROS, a decrease in sperm count, and altered sperm morphology in rats exposed to 900 MHz 3G mobile phone radiation (SAR = 0.26 W/kg) 2 hr./day for 45 days [61]; decreased sperm quality in rats in which local exposure of the scrotum to 2575–2635 MHz 4G smartphone time division LTE radiation occurred for 1 min over 10 min intervals 6 hr./day for 150 days [62]; impaired testicular development at 35 days of age in male offspring of pregnant rats that were exposed to 2.45 GHz RFR (SAR = 1.75 W/kg) 2 hr./day throughout pregnancy [63]; decreased sperm motility in mice exposed to 905 MHz RFR (SAR = 2.2 W/kg) 12 hr./day for 5 weeks, and increased ROS formation and DNA fragmentation after 1 week of exposure [64]. Although negative studies have also been reported, it is important to remember that the outcome of experimental studies can be affected by differences in exposure conditions, including the frequency, modulation, polarization, stray electromagnetic fields, local SAR, duration of exposure, and analytical methods [43, 44].

Although the mechanism of testicular effects from exposure to non-thermal levels of RFR is not fully known, numerous studies in rats and mice, and in human sperm have found associations between negative effects on fertility parameters and increases in ROS and/or DNA damage [48, 51, 57, 58, 60, 61, 64–68]. Thus, the adverse effects of RFR on sperm quality are likely due in large part to induced generation of ROS.

- **Assumption 2**) *RF radiation is incapable of causing DNA damage other than by heating; there is no mechanism for non-thermal DNA damage.*

In 2009, ICNIRP [16] claimed that “low energy photons of RF radiation are too weak to affect ionization or cause significant damage to biological molecules such as DNA, under ordinary circumstances.” However, DNA damage and other genotoxic effects have been observed in numerous studies of low intensity RFR in animal models and in humans. For example, the NTP study found statistically significant increases in DNA damage in brain cells of exposed rats and mice compared to sham controls [18, 19, 69], and Akdag et al. [70] found statistically significant increases in DNA damage in hair cells in the ear canal among 30 to 60 year-old men who used mobile phones for 10 years for 0–30 min/day, 30–60 min/day, or greater than 60/min/day compared to people who did not use mobile phones. In the latter study, the extent of DNA damage increased with increasing daily exposure duration. In a review of published studies on genetic effects of ELF- and RF-EMF, Lai [71] listed more than 150 studies in which non-thermal exposures to RFR produced increases in DNA damage, chromosome aberrations, or micronuclei formation.

In addition, it is well established that DNA damage can also be caused by indirect processes, such as by the generation of reactive oxygen species (ROS), and numerous studies have demonstrated DNA damage at exposures below the putative threshold SAR of 4 W/kg. More than 120 published studies have demonstrated oxidative effects associated with exposure to low intensity RFR (Additional file 1: Appendix 1). An analysis of experimental studies on molecular effects of low intensity RF radiation (RFR) in biological systems found that the majority (93 of 100 studies) demonstrated the induction of oxidative effects [72]. More recent studies (from 2017) revealed that all 30 relevant publications (100%) detected significant oxidative effects under low intensity RFR exposures, and most of these studies used modulated RFR from wireless communication devices.

Increased production of ROS in living cells may be caused by weak magnetic fields altering recombination rates of short-lived radical pairs generated by normal metabolic processes leading to changes in free radical concentrations [73], or by low intensity extremely low frequency (ELF) EMFs resulting in alterations in voltage-gated ion channels in cell membranes causing changes in cation flow across membranes [74]. These mechanisms apply to both ELF-EMFs and to RFR modulated by pulsed fields at extremely low frequencies. Other biophysical mechanisms by which non-thermal RF-EMF can cause biological effects through interactions with normal cellular processes have been described [75].

Increasing NADH oxidase activity is another mechanism by which RFR can increase ROS production. NADH oxidases, which are membrane-associated enzymes that catalyze one-electron reduction of oxygen to superoxide radical using NADH as the electron donor, have been identified as primary mediators of RFR interactions in cellular systems [76]. A significant (3-fold) increase in the activity of NADH oxidase was measured in purified plasma membranes from HeLa cells exposed to 875 MHz for 5 or 10 min at a power density of 200 μW/cm^2^. This exposure intensity is significantly lower than the ICNIRP [5] safety limit.

The major source of ROS in living cells is the mitochondrial electron transport chain, where leakage of electrons generates superoxide radicals due to the partial reduction of oxygen [77]. A dose-dependent effect of 1.8 GHz modulated RFR exposure (SAR = 0.15 and 1.5 W/kg) on mitochondrial ROS production was detected in mouse spermatogonial germ cells [65]. Exposure of quail embryos to extremely low intensity modulated RFR (GSM 900 or 1800 MHz, 0.25 or 0.32 μW/cm^2^) during the initial days of embryogenesis resulted in a robust overproduction of superoxide radical and nitrogen oxide in mitochondria of embryonic cells [78, 79]. Thus, multiple mechanisms for the increased production of ROS by low intensity RF radiation have been demonstrated.

Numerous studies have been published on mutagenic effects of low intensity RF-EMFs, especially studies that identified increases in levels of a specific marker of oxidative DNA damage and a risk factor for cancer, 8-hydroxy-2′-deoxyguanosine (8-OHdG) [58, 60, 78–84]. For example, the level of 8-OHdG in human spermatozoa was increased significantly after in vitro exposure for 16 hr. to 1.8 GHz at a power level of 2.8 W/kg and correlated with levels of ROS generation [58]. Likewise, exposure of quail embryos *in ovo* to GSM-modulated 900 MHz of 0.25 μW/cm^2^ for 1.5, 5, or 10 days was sufficient to produce a significant, two-threefold, increase in 8-OHdG levels in embryonic cells [79]. Umbilical cord blood and placenta tissue samples obtained after delivery from women who used mobile phones during pregnancy had significantly higher levels of oxidative stress parameters, including 8-OHdG and malondialdehyde, compared to cord blood and placental tissue from women who did not use mobile phones during pregnancy [85]. In addition, DNA damage, analyzed by the comet assay, was increased significantly in cord blood lymphocytes obtained from women who used mobile phones during pregnancy compared to cord blood lymphocytes obtained from women who did not use mobile phones.

As low intensity RF radiation does not have sufficient energy to ionize DNA molecules, and as increased production of ROS in living cells due to RF-EMF exposures has been reliably documented, an indirect effect of this type of radiation is the formation of oxidative damage to DNA. The most aggressive form of ROS that can cause oxidative DNA damage is the hydroxyl radical; this reactive oxygen species can be generated from superoxide radical and hydrogen peroxide [86], which may be produced in living cells exposed to low intensity RF radiation. Ultraviolet radiation (UVR, encompassing UVA, UVB, and UVC), which is classified by IARC as “carcinogenic to humans”), can also cause indirect DNA damage by generating ROS [87]. Thus, both RFR and UVR, which can similarly induce oxidative DNA damage, can increase cancer risk by a similar mechanism.

Increased production of ROS and depletion of antioxidant capacity in living cells exposed to low intensity RF radiation can result in oxidative DNA damage. Induction of oxidative stress, which is a key characteristic of many human carcinogens [88], including UVR and asbestos, can also lead to genotoxicity and carcinogenicity of non-ionizing RF radiation without causing direct DNA damage.

- **Assumption 3**) *Two to seven exposures to RF radiation for up to 1 hour duration are sufficient to exclude adverse effects for any duration of exposure including chronic exposures.*

The behavioral studies in 8 male rats and 5 male monkeys that served as the basis for the exposure limits to RF radiation adopted by the FCC and ICNIRP involved 2 to 7 exposure sessions of 40-minute duration for rats [10] and 3 exposure sessions of 60-minute duration for monkeys at each power density [11]. Additional support for the threshold SAR of 4 W/kg in the frequency range of 100 kHz to 6 GHz came from behavioral studies conducted in rats and monkeys by D’Andrea et al. [89, 90]. However, D’Andrea et al. [91, 92] also reported that exposure of rats to continuous wave 2450 MHz RFR for 14 or 16 weeks caused significant differences in behavioral activity between sham-exposed rats and RFR-exposed rats at mean SARs of 0.7 W/kg and at 1.23 W/kg, indicating that 4 W/kg is not a threshold SAR with extended exposure durations. Since that time many studies have shown that responses to non-thermal RFR depend on both exposure intensity and exposure duration [93]. Importantly, the same response was observed with lower exposure intensity but prolonged exposure duration as at higher exposure intensity and shorter duration [94].

Recognizing that the exposure limits do not address potential health effects after long-term exposures to RF radiation emitted from wireless devices that people are experiencing, the FDA [17] nominated RF radiation to the NTP for chronic toxicology and carcinogenicity studies out of concern that “existing exposure guidelines are based on protection from acute injury from thermal effects of RFR exposure, and may not be protective against any non-thermal effects of chronic exposures.” Adverse health effects noted in Assumption #1, including cardiomyopathy, carcinogenicity, sperm damage, and neurological effects, as well as the human epidemiology studies to be described in Assumption #6, occurred with much longer exposures to RF radiation than the exposure durations used in the acute studies in rats [10] and monkeys [11]. Consequently, the acute behavioral exposure studies that served as the basis for exposure limits to RF radiation established by the FCC and ICNIRP are inadequate to identify and characterize adverse effects of RF radiation after longer exposure durations. Neither the exposure limits established in the 1990s by the FCC [4] or by ICNIRP [9], nor those reaffirmed more recently by these groups [3, 5] address health risks associated with long-term exposure to RF radiation.

- **Assumption 4**) *No additional effects would occur from RF radiation with co-exposure to other environmental agents.*

The current FCC/ICNIRP exposure limits do not take into consideration interactive effects of RF radiation with other environmental agents even though such effects have been documented. Interactions of RF radiation with other agents may result in antagonistic or synergistic effects, i.e., effects that are greater than the sum of each agent alone.

In the International Agency for Research on Cancer (IARC) evaluation of the carcinogenicity of RF-EMF [44], the expert working group noted that 4 of 6 co-carcinogenesis studies available at that time showed increased responses with exposure to RF-EMF. One of those studies reported co-carcinogenic effects of UMTS-modulated RF radiation at 4.8 W/m^2^ in the liver and lung of mice that had been treated with the carcinogen ethylnitrosourea (ENU) in utero [95]; the incidence of liver and lung cancers were increased in mice exposed to ENU plus RF radiation compared to cage controls, sham controls and ENU alone. After the IARC evaluation, Lerchl et al. [96] replicated the experimental design of Tillmann et al. [95] by exposing mice to RF-EMF at whole-body SAR levels of 0 (sham), 0.04, 0.4, and 2 W/kg. Significant increases in lung adenomas and/or liver carcinomas were observed at all exposure levels. Lerchl et al. [96] concluded that their “findings are a very clear indication that tumor-promoting effects of life-long RF-EMF exposure may occur at levels supposedly too low to cause thermal effects.” Thus, the reproducibility of the tumor-promoting effects of RFR at non-thermal exposure levels has been demonstrated.

Other examples of reported synergistic effects include the following study results. Synergistic effects on damage to human lymphocytes were observed with co-exposure to RFR (1.8 GHz RFR, SAR 3 W/kg) and 2 different mutagens, namely, mitomycin C or 4-nitroquinoline-1-oxide [97], or with co-exposure to ultralight (UVC) light [98]. A synergistic effect was found on DNA damage in human blood cells exposed to 2450 MHz radiation (5 mW/cm^2^) and then exposed to mitomycin C [99]. A potentiation effect on DNA damage was observed in cultured mammalian cells exposed to CDMA-modulated 835 MHz RF-EMF (SAR = 4 W/kg) and the clastogens cyclophosphamide or 4-nitroquinoline-1-oxide [100]. Gene expression was altered in neuronal and glial cells of rats pre-treated with lipopolysaccharide, a neuroinflammatory agent, and then exposed to 1800 MHz GSM modulated radiation (SAR = 3.22 W/kg) for 2 hr. [101]. In rats pre-treated with picrotoxin, a chemical that induces seizures, exposure to pulse-modulated 900 MHz GSM-modulated RF radiation of mobile phones increased regional changes in brain activity and c-Fos expression [102, 103].

Exposure limits based on exposure to only RF radiation will result in an underestimation of the true risk and inadequate protection of human health under conditions in which co-exposures to other toxic agents lead to synergistic adverse effects [104].

### B. Factors affecting dosimetry

- **Assumption 5**) *Health effects are dependent only on the time-averaged SAR value; carrier wave modulations, frequency, or pulsing do not matter except as they influence the SAR.*

The FCC’s and ICNIRP’s exposure limits to RFR are based on SARs for frequencies up to 6 GHz and on power densities for frequencies between 6 GHz and 300 GHz averaged over 6-minute or 30-minute intervals for local areas and whole-body exposures [3, 5]. However, time-averaged dosimetry does not capture the unique characteristics of modulated or pulsed RFR. For example, GSM modulation may involve as many as 8 voice channels with a duration of 0.577 msec for each channel. Thus, the exposure from GSM modulation can be 8-times higher during each time slot pulse compared to exposure to a continuous wave at equivalent time-averaged SARs. Also, as noted under assumption #14, repetitive pulses of data in bursts with short exposures to 5G can cause localized temperature spikes in the skin [105]. The impact of pulsed radiation on biological activities at the molecular or cellular levels is not taken into consideration with time-averaged dosimetry.

Another issue not addressed by time-averaged dosimetry is the importance of low frequency modulations on biological systems. As discussed under assumption #2, increased production of ROS in living cells and DNA damage have been demonstrated with exposure to low frequency modulations of radiofrequency carrier waves [106]. Exposure limits based on time-averaged SAR dosimetry or power density, without consideration of the impact of amplitude or frequency modulations, do not adequately address potential health effects of real-world exposures to RFR. There is ample evidence that various effects of RFR exposure depend on carrier wave modulations, frequency, or pulsing [43, 107, 108]. In contrast to ICNIRP/FCC, the IARC monograph on RFR carcinogenicity noted that RFR effects may be influenced by such exposure characteristics as duration of exposure, carrier frequency, type of modulation, polarization, exposure intermittence, and background electromagnetic fields [44].

### C. Human brain tumor risk

- **Assumption 6)** *The multiple human studies that find associations between exposure to cell phone RF radiation and increases in brain tumor risk are flawed because of biases in the published case-control studies, and because brain cancer rates have remained steady since the time that use of wireless communication devices became widespread*.

Although claims have been made that “current limits for cell phones are acceptable for protecting the public health” because “even with frequent daily use by the vast majority of adults, we have not seen an increase in events like brain tumors” [109], the SEER (Surveillance, Epidemiology, and End Results Program) database shows an annual decrease of 0.3% for all brain tumors, but an increase of 0.3% per year for glioblastoma in the US between 2000 and 2018 (https://seer.cancer.gov/explorer/). Most concerning was that the annual increase for glioblastoma was 2.7% per year for people under 20 years of age. In addition, Zada et al. [110] reported that the incidence of glioblastoma multiforme (GBM) in the frontal lobe, temporal lobe, and cerebellum increased in the US between 1992 and 2006, and Philips et al. [111] likewise reported a statistically significant increasing incidence of GBM in the frontal and temporal lobes of the brain in the UK during 1995–2015. In Sweden, rates of brain tumors in the Swedish National Inpatient Register and the Swedish Cancer Register increased from 1998 to 2015 [112]. In addition, it should be realized that cumulative exposure, side-of-head use, and latency for tumor formation from RFR are not fully captured in national cancer registries. Thus, the claim that trends in brain cancer incidence rates have not increased since mobile phones were introduced is both wrong and misleading. The specificity of effect needs to be factored into such trend analyses.

Case-control studies, using sound scientific methods, have consistently found increased risks with long-term, heavy mobile phone use for brain tumors of the glioma type and acoustic neuroma. This association was evaluated at IARC in 2011 by 30 expert participants who concluded that radiofrequency (RF) radiation is a “possible” human carcinogen [44]. In contrast, the much-cited Danish cohort study on ‘mobile phone users’ [113] was disregarded by IARC due to serious methodological shortcomings in the study design, including exposure misclassifications [44, 114].

Results of meta-analyses of glioma risk and acoustic neuroma from Swedish case-control studies conducted by Hardell and coworkers [115, 116], the 13-nation Interphone study [117], and the French study by Coureau et al. [118] are shown in Table 1 as odds ratios (OR) with 95% confidence intervals. For glioma on any location in the head, a statistically significant increase of nearly two-fold was found, while for ipsilateral mobile phone use (tumor and phone use on the same side of the head) the risk was increased by 2.5-fold. These ORs are based on the groups in each study with the highest category of cumulative call time, which were ≥ 1640 hr. in the Interphone study [117, 119] and the Swedish studies [115, 116], and ≥ 896 hr. in the study by Coureau et al. [118]. Decreased survival among glioma cases, especially astrocytoma grade IV, was associated with long-term and high cumulative use of wireless phones [120]. Increased risk for the mutant type of *p53* gene expression in the peripheral zone of astrocytoma grade IV was associated with use of mobile phones for ≥3 hours a day. Increase in this mutation was significantly correlated with shorter overall survival time [121].

**Table 1:** Odds ratios (OR) with 95% confidence interval (CI) for glioma and acoustic neuroma in case-control studies in the highest category for cumulative mobile phone use in hours^a^

	Glioma				Acoustic neuroma			
	All		Ipsilateral		All		Ipsilateral	
	OR	95% CI	OR	95% CI	OR	95% CI	OR	95% CI
Interphone [117, 119] Cumulative use ≥1640 hr	1.40	1.03–1.89	1.96	1.22–3.16	1.32	0.88–1.97	2.33	1.23–4.40
Coureau et al. [118] Cum use ≥896 hr	2.89	1.41–5.93	2.11	0.73–6.08				
Hardell et al. [115, 116] Cumulative use ≥1640 hr	2.13	1.61–2.82	3.11	2.18–4.44	2.40	1.39–4.16	3.18	1.65–6.12
**Meta-analysis** longest cumulative use	**1.90**	**1.31–2.76**	**2.54**	**1.83–3.52**	**1.73**	**0.96–3.09**	**2.71**	**1.72–4.28**

^a^ Note Hardell et al. [115, 116] also assessed use of cordless phones

For acoustic neuroma, risk was significantly increased with cumulative exposure and ipsilateral use by 2.7-fold. A random effects model, which was based on a test for heterogeneity, was used for the meta-analyses of these published studies. Tumor volume of acoustic neuroma increased per 100 hr. of cumulative use of wireless phones in the Swedish study and years of latency, indicating tumor promotion [115].

Other case-control studies of mobile phone use also reported increased risk of acoustic neuroma [122–124]. Those studies were not included in the meta-analysis because data on cumulative mobile phone use with numbers of cases and controls were not given or there were other shortcomings. It is also noteworthy that tumor risks were increased in subsets of the Interphone study; for example, there was nearly a 2-fold increase in the risk of acoustic neuroma for ≥10 y and ipsilateral use among the North European countries that participated in the Interphone study [125].

Claims have been made that associations between increases in brain cancer risk and exposure to cell phone RF radiation in the published case-control studies may be attributable to recall and/or selection biases [5, 109]. However, a re-analysis of the Canadian data that was included in the Interphone study showed that there was no effect on the risk of glioma after adjustments were made for selection and recall biases [126]. Odds ratios (OR) for glioma were increased significantly and to a similar extent when comparing the highest quartile of use to those who were not regular users whether or not adjustments for biases were made. In addition, Hardell and Carlberg [116] showed that the risk for glioma with mobile phone use was increased significantly even when compared to the risk for meningioma. Because risk of meningioma was not increased significantly, this tumor response could not be attributed to recall bias. Clearly, selection and recall biases do not explain the elevated brain tumor risk associated with the use of mobile phones. Thus, epidemiological evidence contradicts the opinions of the FCC and ICNIRP on brain tumor risk from RF radiation.

It should also be noted that the thyroid gland is a target organ for RFR from smartphones. A case-control study on mobile phone use suggested an increased risk for thyroid microcarcinoma associated with long-term cell phone use [127]. Peripheral lymphocyte DNA obtained from cases and controls was used to study genotype-environment interactions. The study showed that several genetic variants based on single nucleotide polymorphisms (SNPs) increased the risk of thyroid cancer with mobile phone use [128]. Increasing incidence of thyroid cancer in the Nordic countries, especially over the last two decades, has also been reported [129, 130]. In addition, a recent case-control study found significant increases in breast cancer risk among Taiwanese women based on their use of smartphones and distance between the breast and placement of their smartphone [131].

### D. Individual variations in exposure and sensitivity to RF-EMF

- **Assumption 7)** *There are no differences among individuals, including children, in the absorption of RF-EMF and susceptibility to this radiation.*

Differences between children and adults regarding the absorption of radiofrequency electromagnetic fields when mobile phones are operated close to the head have been demonstrated and widely documented [132–137]. The main factors accounting for these dissimilar absorption rates include differences in anatomy, tissue dielectric properties, and physiology. Through finite-difference time-domain (FDTD) simulations, employing detailed computational anthropomorphic models, it is possible to find differences relating to anatomy and to dimensions of the head.

Since EMF penetration into human tissues can be in the order of a few centimeters, depending on the wavelength, the inner tissues in the brain clearly will receive a significantly higher dose in the smaller heads of children compared to adults, despite the total absorption and the peak spatial SAR (psSAR) calculated across the whole head varying by smaller amounts [132, 133, 138]. Fernández et al. [136] estimated that the cell phone radiation psSAR in the hippocampus was 30-fold higher in children compared to adults, while the psSAR in the eyes was 5-fold higher in children; these differences were due largely to closer proximity to the cell phone antennas. The thinner dimensions of children’s skulls also contribute to this difference [135], resulting in a psSAR around 2-fold higher in children’s brains [134–137, 139] compared to adults.

Additionally, tissues of young mammals have higher conductivity and electrical permittivity than those of mature animals [140]. This also contributes to greater EMF penetration and absorption, resulting in further increases in the psSAR. The psSAR in the skull bone marrow of children was estimated to increase by 10-fold due to higher conductivity in this tissue [137]. Distance between the mobile device and the body tissues is important in characterizing tissue dosimetry. The National Agency ANFR of France recently released cell phone SAR test data for 450 cell phones. Ten gram psSARs increased by 10–30% for each millimeter of proximal placement of the cell phone to the planar body phantom (http://data.anfr.fr/explore/dataset/das-telephonie-mobile/?disjunctive.marque&disjunctive.modele&sort=marque).

Finally, it is important to note that simulations of tissue dosimetry consider only the physical parameters of the tissues; they do not consider biological processes occurring in living tissues. While children are growing, developing organs and multi-organ systems are more susceptible to adverse effects of environmental agents; finite-difference time-domain (FDTD) simulations do not address differences in organ or system susceptibility for exposures occurring during child development.

- **Assumption 8)** *There are no differences among individuals in their sensitivity to RF radiation-induced health effects.*

All life is “electrosensitive” to some degree as physiological processes are dependent on both subtle and substantial electromagnetic interactions at every level, from the molecular to the systemic. Responses to multiple types of electromagnetic exposure reveal that there is a far broader range of EMF sensitivity than previously assumed, and subgroups of extremely hypersensitive subjects exist [141–151]. Given the adverse health effects noted in Assumption #1, including cardiomyopathy, carcinogenicity and neurological effects, the acute, conscious symptoms manifesting in some individuals should not be unexpected. The term currently and most frequently used within the medical profession to describe those who are acutely, symptomatically sensitive to non-ionizing radiation exposures is Electromagnetic Hypersensitivity (EHS).

EHS is a multisystem, physical response characterized by awareness and/or symptoms triggered by EMF exposures. Common symptoms include (but are not limited to) headaches, dizziness, sleep disturbance, heart palpitations, tinnitus, skin rashes, visual disturbance, sensory disturbance, and mood disturbance [152, 153]. These symptoms are reported in response to even extremely low intensity (orders of magnitude below current safety levels) EMFs of multiple types (in terms of frequency, intensity and waveforms). Commonly noticed triggers of frequent and persistent EHS symptoms are pulse-modulated RF emissions, modulated at extremely low frequencies. Common triggering sources include mobile phones, DECT cordless landlines, Wi-Fi/Bluetooth-enabled computers, Wi-Fi routers, smart meters, base station antennas, and household electrical items. EMF avoidance/mitigation is found to be the most effective way to reduce symptoms [154].

Guidelines for EHS diagnosis and management have also been peer-reviewed and concur that the mainstay of medical management is avoidance of anthropogenic electromagnetic fields [152, 155, 156]. Case histories detailing clinical presentations, EMF measurements and mitigation are also published [157], and biomarkers including elevated markers of oxidative stress, inflammatory markers and changes in cerebral blood flow continue to be explored [152].

EHS has been proven to be a physical response under blinded conditions [145, 151, 158, 159] and, in addition to these studies, acute EMF-induced changes in cognition, behavior, and physiology reactions have been observed in studies involving animals [27, 30, 160–172]; plus further references under Assumption 13), which cannot be biased by media-cultivated fears. These studies provide further evidence which invalidates the nocebo response (physical symptoms induced by fear) as causal regarding symptoms.

It should not be expected that all provocation studies will reliably demonstrate adverse reactions; however, suggestions that the nocebo response may cause EHS symptoms were claimed from provocation studies which failed to show a relationship between the EMF exposure and the reported symptoms [173]. The failures of these studies are explainable given the very poor methodology in the majority of them. There were failures to account for a multitude of essential factors that must be tailored to the individual, such as variable symptom onset and offset, the necessity for adequate washout periods, specificity of trigger frequencies and intensities, requirement for complete EMF hygiene during sham exposures, requirement for life-like exposures (e.g., pulse-modulated information-carrying waves), etc. For example, it has been shown that various frequency channels from GSM/UMTS mobile phones affect the same human cells differently [174–177]. Similarly, EHS has been shown to be frequency dependent [151]. As noted above, meaningful provocation studies need to take into consideration multiple physical parameters of exposure, including frequency, modulation, duration of exposure, and time after exposure [155]; however, most provocation studies that have failed to establish causative connection between RFR exposure and EHS symptoms [173] used only one or two conditions with short-term exposures.

There are many issues with the nocebo response as a cause of EHS, not least of which is also the absence of the required temporal link. For the nocebo response to be the cause of EHS, awareness and concern of negative health impacts from EMFs must precede symptoms. But, in the majority of EHS persons this is not the case [178]. As public risk communication improves, this will no longer be verifiable; however, this has been importantly observed at the only point in time when it could have been – prior to generalized awareness of health detriments from non-ionizing radiation (NIR).

While recognizing that some vulnerable groups may be more susceptible to effects of NIR exposure, ICNIRP [179] acknowledged that their guidelines may not safely accommodate these sensitive subgroups:

“Different groups in a population may have differences in their ability to tolerate a particular NIR [Non-Ionizing Radiation] exposure. For example, children, the elderly, and some chronically ill people might have a lower tolerance for one or more forms of NIR exposure than the rest of the population. Under such circumstances, it may be useful or necessary to develop separate guideline levels for different groups within the general population, but it may be more effective to adjust the guidelines for the general population to include such groups. Some guidelines may still not provide adequate protection for certain sensitive individuals nor for normal individuals exposed concomitantly to other agents, which may exacerbate the effect of the NIR exposure, an example being individuals with photosensitivity”.

In 2020, ICNIRP [23] also noted that biological effects are not easily discernible from adverse health effects, and that their guidelines:

“…are not intended to protect against biological effects as such (when compensatory mechanisms are overwhelmed or exhausted), unless there is also an associated adverse health effect. However, it is not always easy to draw a clear distinction between biological and adverse health effects, and indeed this can vary depending on individual susceptibility to specific situations. An example is sensory effects from nonionizing radiation exposures under certain circumstances, such as a tingling sensation resulting from peripheral nerve stimulation by electric or magnetic fields; magnetophosphenes (light flickering sensations in the periphery of the visual field) resulting from stimulation of the retina by electric fields induced by exposure to low-frequency magnetic fields; and microwave hearing resulting from thermoelastic waves due to expansion of soft tissues in the head which travel via bone conduction to the inner ear. Such perceptions may sometimes lead to discomfort and annoyance. ICNIRP does not consider discomfort and annoyance to be adverse health effects by themselves, but, in some cases, annoyance may lead to adverse health effects by compromising well-being. The exposure circumstances under which discomfort and annoyance occur vary between individuals”.

Trivializing “discomfort” which is the pre-cursor to pain is not in keeping with WHO recommendations quoted by the same ICNIRP [23] document: “Health is a state of complete physical, mental and social well-being and not merely the absence of disease or infirmity.”

Discomfort is a sign that an organism is experiencing something which is compromising optimal health and although in some cases this can be trivial and reversible, in other cases it may not be reversed. There is an extremely broad range of both pain tolerance and also of pain perception among humans, and to achieve meaningful preventative health care, “discomfort” must be taken seriously and mitigated whenever possible. This is especially true in this case where symptoms such as headaches are being reported in response to mobile phone exposures at the same time as increased brain tumor risk is noted from those same exposures (see Assumption 6).

In reality, people with EHS are reporting far more serious health disruption than “discomfort” or “annoyance” and in some cases these symptoms are disabling [180, 181]. Increasingly, EHS is being recognized as a disability by national courts in France, Sweden, and Spain, which amplifies the requirement for safety guidelines that are deliberately accommodating to this more susceptible group [180].

### E. Applied safety factors for RF-EMF-RF workers and the general population

- **Assumption 9)** *A 50-fold safety factor for whole body exposure to RF radiation is adequate for protecting the general population to any health risks from RF radiation.*

Public health agencies in the US and worldwide apply multiple uncertainty factors to health effects data to establish exposure levels that are considered safe for the great majority of exposed populations [182–184]. Although guidelines for the use of uncertainty factors were developed for chemicals, they are also pertinent to other toxic agents, such as RFR. The uncertainty factors needed for toxic effects of RFR based on studies that demonstrate a no-observed-adverse-effect level (NOAEL) in experimental animals include:

1. Animal-to-human extrapolation. When data are based on studies in experimental animals, a factor of 3–10 is applied (for potential species differences in tissue dosimetry and response) unless there are convincing data demonstrating equivalent sensitivity in animals and humans. However, there is no evidence showing that humans are equally or less sensitive to RFR than animals that were used in studies from which exposure limits were established by the FCC and ICNIRP.
2. Adjustment for human variability. A second factor of 10 is used to account for interindividual variability in susceptibility (for instance, due to differences in age, sex, genetic variation, pre-existing diseases) to the toxic agent among the general population. It has been recognized that a factor of 10 for human variability is likely inadequate for sensitive subpopulations and may require an additional adjustment.
3. Extrapolation from short-term studies to lifetime exposure. An additional factor of 10 is applied for short-term studies, such as those used to establish exposure limits to RF radiation, to provide lifetime protection from chronic exposure. This is of particular importance considering the remarkably short periods over which RFR toxicity was originally assessed [10, 11].
4. Database insufficiencies. Finally, an uncertainty factor of 3-to-10 is applied for database inadequacy, i.e., for incomplete characterization of an agent’s toxicity. The behavioral studies [10, 11] that were used to establish the FCC and ICNIRP exposure limits to RFR do not provide a full characterization of the effects of this type of radiation nor did they identify the most sensitive adverse effect of RFR exposures.

Basing exposure limits to RFR on the behavioral studies in rats and monkeys [10, 11, 90, 91] would require the application of a composite uncertainty factor of about 900 to 10,000 to be consistent with approaches used by public health agencies to establish protective exposure limits for workers and the general population. Based on the size of the needed uncertainty/safety factor, the data sets used by the FCC and ICNIRP are clearly inadequate to establish RF exposure limits with reasonable confidence. The arbitrarily selected safety factors of 10 for workers and 50 for the general population by the FCC and ICNIRP are woefully inadequate for protecting exposed populations.

When uncertainty/safety factors are applied to a misrepresented threshold exposure value for adverse effects, the resulting level does not provide assurance of health protection for the general population exposed to that agent. Studies cited above [18, 22, 91, 92, 96] show that the whole-body SAR of 4 W/kg is not a threshold level for adverse effects caused by RFR. In a recent quantitative analysis of various adverse health effects from the NTP study, Uche and Naidenko [185] showed that the permissible whole-body SAR of 0.08 W/kg (based on a 50-fold reduction of the assumed threshold SAR of 4 W/kg) was 20–40-fold higher than health protective SAR values derived by benchmark dose modelling of NTP data for cardiomyopathy (following application of 10-fold safety factors for interspecies and intraspecies variability). The approaches used by these authors are consistent with methodologies recommended by the US Environmental Protection Agency for quantifying health risks for toxic and carcinogenic environmental agents [1, 182]. Thus, a 50-fold reduction of the assumed threshold whole-body SAR of 4 W/kg is inadequate to protect the health of the general population from exposure to RF radiation.

- **Assumption 10)** *A 10-fold safety factor for whole body exposure to RF radiation is adequate for protecting workers to any health risks from RF radiation.*

When RFR exposure limits were implemented in 1997, the rationale given for the difference in safety factors for the general population (50-fold) and for workers (10-fold) was “based on the exposure periods of the two populations, rounded to one digit (40 work hours per week/168 hours per week = ~0.2)” [6]. In addition to differences in exposure periods between workers and the general population, ICNIRP rationalizes the appropriateness of the lower safety factor for workers because “occupationally-exposed individuals can be considered a more homogeneous group than the general population,” they are, “in general, relatively healthy adults within a limited age range,” and “occupationally-exposed individuals should be operating under controlled conditions and be informed about the risks associated with non-ionizing radiation exposure for their specific situation and how to reduce these risks” [23]. In contrast, “the general public are, in most cases, unaware of their exposure to non-ionizing radiation and, without education, cannot reasonably be expected to take precautions to minimize or avoid any adverse effects of exposure.”

The assumption that workers are trained in understanding health risks associated with exposure to RFR and in mitigating those risks to the greatest possible degree is not correct because neither the FCC nor the ICNIRP guidelines recognize any health effects from RFR at SARs below 4 W/kg, and the exposure limits authorized by the FCC and ICNIRP do not consider health effects from long-term exposures [3, 5]. The only health effect addressed by the FCC and ICNIRP is tissue damage due to excessive heating from acute exposures. Thus, the 10-fold reduction from the threshold whole-body SAR calculated from acute behavioral studies in rats and monkeys is inadequate for protecting the health of workers exposed long-term to RFR (see comments under assumption #9). There are no data demonstrating the adequacy of this arbitrarily chosen safety/uncertainty factor for occupationally-exposed workers, while on the contrary, excess cancer risks have been associated with exposure to RFR workers who operate radar and communication systems in military and occupational settings [186].

- **Assumption 11)** *Exposure of any gram of cube-shaped tissue up to 1.6 W/kg, or 10 g of cube-shaped tissue up to 2 W/kg, (duration not specified) will not increase the risk of that tissue to any toxic or carcinogenic effects in the general population.*

Tissue dosimetry was analysed in the NTP study of cell phone RF radiation in rats and mice [187]. In rats, whole body exposures during the 10-minute on cycles were 1.5, 3.0, or 6.0 W/kg, and the brain and heart SARs varied from the whole-body SARs by about 7% to under 2-fold for the brain and heart, respectively. A quantitative risk assessment of the NTP tumor incidence data is needed to evaluate organ-specific cancer risk. The FDA [19] nomination to the NTP recognized the need for “large well-planned animal experiments …. to provide the basis to assess the risk to human health of wireless communications devices.” However, more than 3 years after an external peer-review of the NTP studies found “clear evidence of carcinogenic activity,” the FDA [109] has continued to downplay the importance of these findings and avoid conducting a quantitative risk assessment of the tumor data that they (the FDA) originally requested. In contrast to the FDA, Uche and Naidenko [185] analysed the NTP data on cardiomyopathy by a benchmark dose approach and found that the 10% extra risk level for this effect was in the range of a whole-body SAR of 0.2 to 0.4 W/kg. Thus, there is an increased risk (greater than 10%) of developing cardiomyopathy at local tissue SARs below 1.6 or 2.0 W/kg.

The peak spatial specific absorption rate (psSAR), as used by ICNIRP and the FCC, is an inadequate dosimetric of RF radiation at frequencies above 1 GHz. The psSAR is calculated by averaging fixed cubic volumes containing a given amount of mass, and assumes a homogeneous material with a given mass density. The ICNIRP recommendation is to average cubic volumes containing 10 g of tissue (10 g-psSAR), while the FCC recommendation is to average cubic volumes containing 1 g of tissue (1 g-psSAR). Current recommendations limit the use of psSAR to frequencies up to 6 GHz [3, 5].

An evaluation of the utility of using psSAR as a dosimetric parameter at different frequencies ranging from 100 MHz to 26 GHz and with cube sizes ranging from 10 mg to 10 g is shown in Additional file 2: Appendix 2. For the smaller cubes and lower frequencies, averaging in the cube does not underestimate the maximum value on the cube surface, but at higher frequencies the psSAR averaged on larger cubes can be several-fold lower than the psSAR averaged on smaller cubes. For example, at 2.45 GHz, averaging over a 10-g cube underestimates by 4 dB (approximately 2.5-fold) the psSAR averaged in smaller cubes, while for 5.8 GHz, averaging over a 10-g cube underestimates the psSAR by 12 dB (approximately 16-fold) compared with averaging in a 10-mg cube, and by 6 dB (approximately 4-fold) compared with averaging over a 1-g cube. When the frequency is increased, the underestimation of the psSAR averaged in larger cubes (e.g. 10 g or 1 g) compared to smaller cubes (e.g. 100 mg and 10 mg) becomes more pronounced. Considering the 10-g cube, the difference between the psSAR for 5.8 GHz EMF compared to 0.9 GHz EMF is around 7 dB (or approximately 5-fold underestimation). These large differences are due to reduced penetration of EMFs at higher frequencies. Therefore, the ICNIRP’s 10 g-psSAR and FCC’s 1 g-psSAR recommendations do not provide reliable dosimetric parameters to evaluate EMF absorption above 1 GHz.

The SAR averaging over a 10-g cube is also flawed for assessing carcinogenicity because it is too large a volume to focus on stem cells and their important role in carcinogenesis. Human stem cells were more sensitive to RFR exposures from GSM and UMTS mobile phones than lymphocytes and fibroblasts [175]. Instead of a random distribution of targets for carcinogenesis, localized distribution of SAR in smaller volumes is needed to more accurately characterize relationships between SAR and tumor induction. From the point of view of stem cell organization, the volume of SAR determinations may be especially important for setting safety limits for children, because most stem cells and their niches are spatially and temporally transient during brain development [188].

- **Assumption 12)** *Exposure of any gram of cube-shaped tissue up to 8 W/kg, or 10 g of cube-shaped tissue up to 10 W/kg, (duration not specified) will not increase the risk of that tissue to any toxic or carcinogenic effects in workers.*

Based on the analyses of tissue dosimetry in the NTP study [187], organ-specific toxic and carcinogenic effects were observed in rats at local tissue SARs that were much lower than 8 or 10 W/kg [18]. The tissue dosimetry in the NTP study and the inadequacy of the local SAR as specified by ICNIRP and the FCC is described in assumption #9.

### F. Environmental exposure to RF radiation

- **Assumption 13)** *There is no concern for environmental effects of RF radiation or for effects on wildlife or household pets.*

While background levels of RF-EMF are increasing in the environment, including rural remote areas [189], neither the FCC nor the ICNIRP take into consideration effects of this radiation on wildlife. The constant movement of most wildlife species in and out of varying artificial EMF can result in high exposures near communication structures, especially for flying species such as birds and insects. There is a substantial amount of scientific literature on the disrupting effects of RFR on wildlife (e.g., [190–206]).

Many nonhuman species use Earth’s geomagnetic fields for activities such as orientation and seasonal migration, food finding, mating, nest and den building [190]. For example, migratory bird species [191, 192], honeybees [193], bats [194], fish [195–197], and numerous other species sense Earth’s magnetic fields with specialized sensory receptors. Mechanisms likely involved in magneto-reception include magnetic induction of weak electric signals in specialized sensory receptors [198], magneto-mechanical interactions with the iron-based crystal magnetite [194], and/or free-radical interactions with cryptochrome photoreceptors [191, 192]. Each of these sensing processes shows extreme sensitivity to low intensity changes in electromagnetic fields. For a fuller description of the mechanisms by which non-human species use magneto-reception to perform essential life activities see Levitt et al. [190].

The following studies represent a few of the many examples of the disrupting effects of low-level exposures to RF-EMF on magneto-reception and the natural behavior of wildlife. Oscillating magnetic fields have been reported to disrupt the ability of migratory birds to orient and navigate in Earth’s geomagnetic field [199–202]. Garden warblers became disoriented by exposure to a weak oscillating magnetic field of 1.403 MHz at an intensity as low as 2–3 nT [200]. The orientation of European robins that use Earth’s magnetic field for compass orientation was completely disrupted by exposure to electromagnetic noise in the frequency range of 50 kHz to 5 MHz or a broadband noise-modulated ELF covering the range ~ 2 kHz to ~ 9 MHz [199, 201]. RFR in the low MHz range (7.0 MHz of 480 nT or 1.315 MHz of 15 nT) has been shown to disable the magneto-reception avian compass as long as the exposure was present [202].

In addition to effects on migratory birds, Landler et al. [203] found that exposure to a low-level magnetic field (1.43 MHz at an intensity of 30–52 nT) disrupted the natural orientation of juvenile turtles hatched on land. GSM-modulated 900 MHz RF radiation caused ants to lose their visual and olfactory memory for finding food [166]. Navigational abilities of trout were reduced when reared under conditions in which magnetic fields were spatially distorted [204].

Activities of honeybees are also disrupted by exposure to RF radiation. GSM-modulated cell phone radiation (900 MHz) caused a reduction in egg laying by queen bees and depletion of beehive pollen and honey counts [205]. GSM-modulated cell phone radiation (900 MHz) reduced hatching and altered pupal development of honey queen bee larvae [206].

The lack of consideration of chronic low-level RF radiation exposure on wildlife could result in dangerously disruptive effects on fragile ecosystems and on the behavior and survival of species that have long existed in Earth’s natural environment.

### G. 5G (5th generation wireless)

- **Assumption 14)** *No health effects data are needed for exposures to 5G; safety is assumed because penetration is limited to the skin (“minimal body penetration”).*

Fifth generation (5G) wireless communication systems are being deployed worldwide to provide higher data transfer rates with shorter lag times between massive numbers of connected wireless devices. To provide faster transfer of large amounts of data (up to 20 gigabits per second peak data rates), the frequency range for 5G includes millimeter waves (30 to 300 GHz), in addition to carrier frequencies as low as 600 MHz. Extremely high frequency millimeter waves (MMW) that transmit large amounts of data to user devices are directed into narrow beams by line-of-sight transmission with beamforming antennas. Because millimeter waves do not penetrate solid structures such as building materials, hills, foliage, etc., and travel only short distances (a few hundred meters), denser networks of base-stations with massive Multiple Input/Multiple Output (MIMO) transmitters and receivers in millions of small cell towers are being installed on structures such as utility poles. These features can lead to much closer proximity between humans and radiation-emitting antennas, and thereby change individual peak and average exposures to RFR.

For a 5G frequency of 26 GHz, EMF absorption is very superficial, which means that for typical human skin, more than 86% of the incident power is absorbed within the first millimeter. The skin penetration depth was computed as 1 mm based on the electrical conductivity of the skin and its electrical permittivity [5, 207]. This is expected to bring the SAR in this tissue well above the recommended limits (**[**208], and Additional file 2: Appendix 2)**.** This is also expected to be harmful to very small species, such as birds and other small animals (e.g., insects) [209]. It is often claimed that because of its shallow penetration, exposure to high frequency 5G radiation is safe, and that the only effect is tissue heating [210]. However, this view ignores the deeper penetration of the ELF components of modulated RF signals, which are rated on the basis of heat alone, as well as the effects of short bursts of heat from pulsed signals [211, 212]. Within the first 1 mm of skin, cells divide to renew the stratum corneum (a consideration for skin cancer), and nerve endings in the dermis are situated within 0.6 mm (eyelids) to 3 mm (feet) of the surface (a consideration for neurological effects). Ultraviolet light, which exerts its action at a penetration depth of less than 0.1 mm [213, 214] is a recognized cause of skin cancer [87].

The higher the frequency of electromagnetic waves, the shorter the wavelength and the shallower the penetration of energy into exposed people or animals. For example, penetration depth in the human body is about 8 mm at 6 GHz and 0.92 mm at 30 GHz [5]. Because of the minimal depth of energy absorption at frequencies above 6 GHz, the FCC and ICNIRP have based exposure limits on power density instead of on SAR levels. The FCC [3] proposed a general localized power density exposure limit of 4 mW/cm^2^ averaged over 1 cm^2^ and not to exceed 30 minutes for 5G services up to 3000 GHz for the general population, claiming that this exposure is consistent with the peak spatial-average SAR of 1.6 W/kg averaged over any 1 g of tissue at 6 GHz. ICNIRP’s [5] exposure limits for 5G are an absorbed power density of 200 W/m^2^ (0.2 W/cm^2^) averaged over 4 cm^2^ and a 6-minute interval for frequencies up to 30 GHz, and 400 W/m^2^ (0.4 mW/cm^2^) averaged over 1 cm^2^ and a 6-minute interval for frequencies of 30 GHz to 300 GHz.

Because of its minimal penetration, exposure to 5G radiation results in higher energy intensity on the skin and other directly-exposed body parts, such as the eye cornea or lens. However, the skin, which is the largest organ in the human body, provides important functions such as acting as a protective physical and immunological barrier against mechanical injury, infection by pathogenic microorganisms, and entry of toxic substances. In addition, skin cancers, including basal cell carcinomas and squamous cell carcinomas, are the most prevalent human cancers, while melanomas are highly metastatic and increasing in prevalence. Although the high incidence of skin cancers are largely attributed to exposure to ultraviolet light, no studies have been reported on the effects of 5G radiation on (i) the skin’s ability to provide protection from pathogenic microorganisms, (ii) the possible exacerbation of other skin diseases, (iii) promotion of sunlight-induced skin cancers, or (iv) initiation of skin cancer by itself. Information is also lacking on the effects of 5G radiation on nervous and immune systems which are also exposed even by the shallower penetration of MMW.

Another important factor is the maximum bandwidth with 5G radiation, which is up to 100 MHz in the frequency range of 450 MHz to 6 GHz, and up to 400 MHz in the ranges from 24 GHz to 52 GHz, compared to previous types of mobile communication where bandwidth is limited to 20 MHz. Because many studies indicated frequency-dependent, non-thermal RF effects from mobile communication RFR [43, 177] and for MMW effects [215, 216], the possibility of effective frequency windows for biological effects would increase with the increased bandwidth of 5G radiation.

Another consideration for effects of 5G exposures on human health is that radiation pulses created by extremely fast data transmission rates have the potential to generate bursts of energy that can travel much deeper than predicted by conventional models [217, 218]. Neufeld and Kuster [105] showed that repetitive pulses of data in bursts with short exposures to 5G can cause localized temperature spikes in the skin leading to permanent tissue damage even when the average power density values were within ICNIRP’s acceptable safety limits. The authors urged the setting of new thermal safety standards to address the kind of health risks possible with 5G technology:

“The FIFTH generation of wireless communication technology (5G) promises to facilitate transmission at data rates up to a factor of 100 times higher than 4G. For that purpose, higher frequencies (including millimetre-wave bands), broadband modulation schemes, and thus faster signals with steeper rise and fall times will be employed, potentially in combination with pulsed operation for time domain multiple access…The thresholds for frequencies above 10 MHz set in current exposure guidelines (ICNIRP 1998, IEEE 2005, 2010) are intended to limit tissue heating. However, short pulses can lead to important temperature oscillations, which may be further exacerbated at high frequencies (>10 GHz, fundamental to 5G), where the shallow penetration depth leads to intense surface heating and a steep, rapid rise in temperature…”

Areas of uncertainty and health concerns with 5G radiation include potential increase in skin cancer rates with (or possibly without) co-exposure to sunlight, exacerbation of skin diseases, greater susceptibility to pathogenic microorganisms, corneal damage or early development of cataracts, testicular effects, and possible resonant-enhanced absorption due to skin structures [219]. One of the complex technical challenges in relation to human exposure to 5G millimeter waves is that the unpredictable propagation patterns that could result in unacceptable levels of human exposure to electromagnetic radiation are not well understood [220]. Although MMW are almost completely absorbed within 1–2 mm in biologically-equivalent tissues, their effects may penetrate deeper in a live human body possibly by affecting signal transduction pathways. Thus, there are too many uncertainties with exposure to 5G to support an assumption of safety without adequate health effects data. There are no adequate studies on health effects from short-term or long-term exposures to 5G radiation in animal models or in humans.

## Discussion

To develop health-based exposure limits for toxic and carcinogenic substances, regulatory agencies typically rely on available scientific evidence about the agent under review. In the mid- and late-1990s when the FCC [4] and the ICNIRP [9] initially established exposure limits for RFR, the prevailing assumptions were that any adverse effects from exposure to RFR were due to excessive heating because non-ionizing radiation did not have sufficient energy to break chemical bonds or damage DNA. However, non-thermal effects of RFR are demonstrated from studies that find different effects with exposure to continuous waves versus pulsed or modulated waves at the same frequency and the same SAR or power density, e.g., [221–226], and from studies that show adverse effects at very low exposure intensities, e.g., [78, 96].

Acute exposure studies conducted in rats and monkeys in the 1980s [10, 11] suggested that an SAR of 4 W/kg could be a threshold dose for behavioral effects. Because this SAR was associated with an approximate increase in body temperature of 1 °C, it was again assumed that no adverse health effects would occur if increases in core body temperature were less than 1 °C. From this putative threshold dose a “safety factor” of 10 was applied for occupational exposures and an additional factor of 5 (50x total) was applied for the general population, resulting in exposure limits in which the whole-body SAR was less than 0.4 W/kg for workers and 0.08 W/kg for the general population. However, realizing that local parts of the body could receive doses of RFR that were 10 to 20 times higher than the whole-body SARs, local peak exposure limits were set by the FCC at SARs 20-times higher than the whole-body SARs, i.e., 8 W/kg averaged over any 1-g of tissue for localized exposures for workers and 1.6 W/kg averaged over any 1-g for the general population [3, 4]. ICNIRP opted for partial body exposures that would not exceed 2.0 W/kg averaged over any 10 g of cube-shaped tissue for the general population [5, 9]. To rationalize the smaller safety factor for workers (10-fold) versus the general population (50-fold), one claim made by ICNIRP [24] is that workers are informed about risks associated with non-ionizing radiation exposure and how to reduce these risks, whereas “the general public are, in most cases, unaware of their exposure to non-ionizing radiation and, without education, cannot reasonably be expected to take precautions to minimize or avoid any adverse effects of exposure.” From a public health perspective, the FCC and ICNIRP should make the public aware of their exposures to RFR and promote precautionary measures to minimize potential adverse effects, especially for children and pregnant women. Eight practical recommendations by the International EMF Scientist Appeal aimed at protecting and educating the public about potential adverse health effects from exposures to non-ionizing EMFs [227] are shown in Table 2.

**Table 2:** Precautionary Measures Recommended by the International EMF Scientist Appeal

1) Priority should be given to protect children and pregnant women	
2) Guidelines and regulatory standards should be strengthened	
3) Manufacturers should be encouraged to develop safer technologies	
4) The public should be fully informed about the potential health risks from electromagnetic energy and taught harm reduction strategies	
5) Medical professionals need to be educated about the biological effects of electromagnetic energy and be provided training on treatment of patients with electromagnetic sensitivity	
6) Governments need to fund training and research on electromagnetic fields and health that is independent of industry	
7) The media should disclose experts’ financial relationships with industry when citing their opinions regarding health and safety aspects of EMF-emitting technologies	
8) Radiation-free areas need to be established, especially for individuals with EHS	

The acute behavioral studies that provide the basis for the FCC’s and ICNIRP’s exposure limits lacked any information on potential effects of RF radiation that can occur after longer durations of exposure, and they did not address effects of carrier wave modulations used in wireless communications. Research on RFR conducted over the past 25 years has produced thousands of scientific papers, with many demonstrating that acute behavioral studies are inadequate for developing health protective exposure limits for humans and wildlife, and that inherent assumptions underlying the FCC’s and ICNIRP’s exposure limits are not valid. First, 4 W/kg is not a threshold SAR for health effects caused by RFR exposures; experimental studies at lower doses and for longer durations of exposure demonstrated cardiomyopathy, carcinogenicity, DNA damage, neurological effects, increased permeability of the blood brain barrier, and sperm damage (see Assumptions 1–3). Multiple robust epidemiologic studies on cell phone radiation have found increased risks for brain tumors (Assumption 6), and these are supported by clear evidence of carcinogenicity of the same cell types (glial cell and Schwann cell) from animal studies. Even studies conducted by D’Andrea et al. [89, 90] before the limits were adopted found behavioral disruption in rats exposed to RFR for 14 or 16 weeks at mean SARs of 0.7 W/kg and at 1.23 W/kg. A combination of exposure duration and exposure intensity would be more appropriate for setting safety standards for exposure to RFR from mobile communication systems including mobile phones, base stations, and WiFi.

More than 120 studies have demonstrated oxidative effects associated with exposure to low intensity RFR (Additional file 1: Appendix 1). DNA damage that has been reported in studies of RFR was most likely caused by induction of oxidative stress, which is a key characteristic of human carcinogens [88], rather than by direct ionization (Assumption 2). The generation of reactive oxygen species has also been linked to DNA damage and the carcinogenicity of UVA radiation [87] and asbestos [228]. Despite the enormous amount of scientific evidence of low-dose effects of RFR, the IEEE [229] maintains that behavioral disruption is still the most sensitive and reproducible effect of RFR. It is this opinion that contributed to the FCC [3] and ICNIRP [5] reaffirming their previous exposure limits to RFR.

Other concerns about the current exposure limits for RFR are that they do not consider potential synergistic effects due to co-exposure to other toxic or carcinogenic agents, the impact of pulsed radiation or frequency modulations, multiple frequencies, differences in levels of absorption or of susceptibility by children, or differences among individuals in their sensitivity to RFR (see Assumptions 4, 5, 7, 8). Currently, children’s cumulative exposures are much higher than previous generations and they continue to increase [230]. ICNIRP [23, 179] acknowledged that their guidelines do not accommodate sensitive subgroups and admit to difficulties separating “biological effects” from “health effects.” Neurological symptoms, some of which are acknowledged by ICNIRP and currently being experienced by persons with EHS, are most certainly non-thermal “health effects” that need to be mitigated by providing environments with reduced exposures to anthropogenic EMF for hypersensitive individuals.

The debilitating effects and restrictions suffered by adults and children with EHS constitutes a contravention of the 2010 Equalities Act, Human Rights Act and other ethical and legal frameworks. Failure to respond and appropriately safeguard this group is already causing preventable morbidity, mortality and economic deficit due to lost workdays, compensations for health damages and increased healthcare costs. Conversely, accommodating this group by, as suggested by ICNIRP [179], acting to ‘adjust the guidelines for the general population to include such groups’ would not only lessen the negative impacts for people with EHS, but would also improve public health more broadly, given the other NIR-related health concerns that are highlighted in this paper.

Basing local tissue exposure limits on 1-g [3] or 10-g [5] cubes substantially underestimates the peak spatial SAR compared to basing local tissue exposure limits on smaller cubes (e.g., 100 mg or 10 mg), and therefore are not reliable dosimetric parameters to evaluate EMF absorption at frequencies above 1 GHz (Assumptions 11, 12). The volumes specified by the FCC and ICNIRP for local tissue SAR limits are too large to focus on stem cells which are important targets for carcinogenesis. To reduce health risks from exposures to RFR, limits for localized distribution of the SAR should be based on 100 mg, or preferably 10 mg cubes.

Another important deficiency raised in this paper is that neither the FCC nor ICNIRP addresses concerns for environmental effects of RFR on wildlife, even though there is extensive literature demonstrating the disrupting effects of RFR on wildlife behavior (Assumption 13).

The arbitrarily selected uncertainty/safety factors applied to the putative threshold SAR for RFR are woefully inadequate for protecting public health (Assumptions 9, 10). Based on the way the US Environmental Protection Agency, the International Council for Harmonization, and the National Institute for Occupational Safety and Health (US NIOSH) apply uncertainty/safety factors to a no-observed-adverse-effect level (NOAEL) in experimental animals [182–184], the safety factor for RFR would be at least 900 to 10,000, which is 18 to 200 times larger than the safety factor recommended by the FCC and ICNIRP for the general population. This large safety factor is based on adjustments for human variability, lifetime exposure from short-term studies, and database insufficiencies that include incomplete characterization of the toxicity of RFR. Clearly, the acute behavioral studies that served as the basis for the current exposure limits for RFR are not suitable for characterizing human health risks associated with long-term exposure to this type of radiation. The NCRP report from 1986 [6] and the ANSI/IEEE document from 1992 [7] recognized that when future studies on biological effects of RFR become available including effects of chronic exposures or evidence of non-thermal interactions there will be a need to evaluate and possibly revise exposure standards. When the FCC [3] and ICNIRP [5] reaffirmed their exposure limits from the 1990s, they dismissed the scientific evidence that invalidated the assumptions that underlie the basis for those exposure limits. An independent re-evaluation of RFR exposure limits based on the scientific knowledge gained over the past 25 years is needed and is long overdue. This evaluation should be performed by scientists and medical doctors who have no conflicting interests and who have expertise in RF-EMF exposure and dosimetry, toxicology, epidemiology, clinical assessment, and risk assessment. Special precautions should be taken to ensure that interpretations of health effects data and the setting of exposure limits for RFR are not influenced by the military or the telecommunications industry. In the meantime, manufacturers should be obliged to develop safer technologies [227].

Finally, we note our concern about the worldwide deployment of 5G communication networks for faster transfer of large amounts of data, but with no adequate health effects studies demonstrating the safety of high frequency millimeter waves. Because of limitations of the penetration and distance of travel of millimeter waves, dense networks of base stations are being mounted on structures such as utility poles in highly populated cities. Also, because the absorption of EMF at frequencies above 6 GHz is minimal, ICNIRP [5] has specified absorbed power density (S_ab_) as the dosimetric parameter for “heating effects” at the higher frequencies. S_ab_ is a function of the incident power density (S_inc_) and the input reflection coefficient (Γ). In near field scenarios, the S_inc_ does not have a singular value; this is largely due to the heterogeneous nature of human body tissues and their relevant parameters (such as the permittivity, equivalent conductivity, mass density), which vary in different body regions and with frequency. Therefore, unless a powerful EMF simulation method together with realistic human models are used, the S_inc_ and the reflection coefficient values would be difficult to accurately estimate, making the resulting S_ab_ unreliable.

The assumption that 5G is safe at the power density limits recommended by ICNIRP (50 W/m^2^ and 10 W/m^2^ averaged over 6 min for occupational and 30 min for public exposures, respectively) because of its minimal penetration into the body does not justify the dismissal of the need for health effects studies prior to implementing 5G networks. The new communication networks will result in exposures to a form of radiation that has not been previously experienced by the public at large (Assumption 14). The implementation of 5G technology without adequate health effects information raises many questions, such as: Will exposure to 5G radiation: (i) compromise the skin’s ability to provide protection from pathogenic microorganisms? (ii) will it exacerbate the development of skin diseases? (iii) will it increase the risk of sunlight-induced skin cancers? (iv) will it increase the risk of damage to the lens or cornea? (v) will it increase the risk of testicular damage? (vi) will it exert deeper tissue effects either indirectly following effects on superficial structures or more directly due to deeper penetration of the ELF components of modulated RF signals? (vii) will it adversely affect wildlife populations? Answers to these questions and others that are relevant to human and wildlife health should be provided *before* widespread exposures to 5G radiation occur, not afterwards. Based on lessons that should have been learned from studies on RFR at frequencies below 6 GHz, we should no longer rely on the untested assumption that current or future wireless technology, including 5G, is safe without adequate testing. To do otherwise is not in the best interest of either public or environmental health.

## Supplementary Information

**Additional file 1: Appendix 1 Table 1.** Studies demonstrating increased oxidative DNA damage and other indicators of oxidative stress at SAR < 4 W/kg.

**Additional file 2: Appendix 2.** On the Inadequacy of the psSAR Dosimetric Parameter at Frequencies above 1 GHz. **Table 1.** Electric permittivity and electric conductivity of the gray matter. **Figure 1.** A block of gray matter radiated by different frequencies. The highlighted cubes are of 10 g, 1 g, 100 mg and 10 mg. **Fig. 2.** A block of gray matter radiated by different frequencies. The highlighted cubes are of 10 g, 1 g, 100 mg and 10 mg. **Fig. 3.** Electric field intensity averaged in each cube for different frequencies: in the left axis, the electric field is in dB and in the right axis the electric field is in V/m normalized to 100 V/m.

## Data Availability

All literature citations are available online.

## Abbreviations

ANSI: American National Standards Institute
CDMA: Code-division multiple access
dB: Decibel
DECT: Digital enhanced cordless technology
EHS: Electromagnetic hypersensitivity
ELF: Extremely low frequency
EMF: Electromagnetic field
FCC: Federal Communications Commission
FDA: Food and Drug Administration
GHz: Gigahertz
GBM: Glioblastoma multiforme brain cancer
GSM: Global system for mobile communication
IARC: International Agency for Research on Cancer
ICNIRP: International Commission on Non-Ionizing Radiation Protection
IEEE: Institute of Electrical and Electronics Engineers
LTE: Long Term Evolution (4G)
MMW: Millimeter wave
NCRP: National Council on Radiation Protection and Measurements
NIR: Non-ionizing radiation
nT: Nanotesla
NTP: National Toxicology Program
8-OHdG: 8-hydroxy-2’deoxyguanosine
psSAR: Peak spatial specific absorption rate
RFR: Radiofrequency radiation
ROS: Reactive oxygen species
SAR: Specific absorption rate
UMTS: Universal mobile telecommunications service (3G)
UVR: Ultraviolet radiation
5G: 5th generation wireless
