# Supplementary material for: Scientific evidence invalidates health assumptions underlying the FCC and ICNIRP exposure limit determinations for radiofrequency radiation: implications for 5G
Source: Environ Health. 2022 Oct 18;21:92. doi: 10.1186/s12940-022-00900-9 (PMC9576312; doi:10.1186/s12940-022-00900-9)
Supplement: Supplementary file 1 — Additional file 1: Appendix 1 Table 1. Studies demonstrating increased oxidative DNA damage and other indicators of oxidative stress at SAR < 4 W/kg. [file 12940_2022_900_MOESM1_ESM.docx]

**Appendix 1**

**Table 1. Studies demonstrating increased oxidative DNA damage and other indicators of oxidative stress at SAR < 4 W/kg**

| **Reference** | **Biological system exposed** | **RFR exposure** | | **Statistically significant effects reported*** |
| --- | --- | --- | --- | --- |
| **In humans** | | | | |
| ([Moustafa et al., 2001](#_ENREF_76)) | Human male body | Cell phone in a pocket in standby position, for 1; 2 or 4 h | | Plasma level of lipid peroxides (LPO) was increased, activities of superoxide dismutase (SOD) and glutathione peroxidase GSH-Px in erythrocytes decreased. |
| ([Garaj-Vrhovac et al., 2011](#_ENREF_36)) | Human whole body | 3; 5.5; 9.4 GHz, pulsed, from radars | | Increased level of malondialdehyde (MDA), decreased level of glutathione (GSH). |
| ([Hamzany et al., 2013](#_ENREF_40)) | Human head/whole body | RFR from cell phone a mean time of 29.6 h/month for 12.5 years | | Increase in all salivary oxidative stress indices. |
| ([Abu Khadra et al., 2014](#_ENREF_1)) | Human male head | GSM 1800 MHz from cell phone, SAR=1.09 W/kg, for 15 and 30 min | | SOD activity in saliva increased. |
| ([Akdag et al., 2018](#_ENREF_5)) | Men, 30-60 years old | Mobile phone radiation: 0-30 min/day, 30-60 min/day or more than 60 min/day | | Comet Assay DNA damage indicators in ear canal hair follicle cells were higher in the RFR exposure groups than in the control subjects. DNA damage increased with the daily duration of exposure. |
| ([Akkam et al., 2020](#_ENREF_7)) | Humans | The use of cell phones, and the presence of a Wi-Fi modem | | A proportional effect on blood glutathione S transferase (e-GST) activities. |
| ([Shaheen et al., 2021](#_ENREF_100)) | Humans / University students | The use of mobile phones | | MDA was higher in high mobile users, negative correlation between emissions from mobile phone and SOD activities. |
| Bektas et al., 2020 | Human umbilical cord blood and placental tissue | Use of mobile phones during pregnancy | | 8-OH-dG, MDA, and DNA damage by comet assay was increased in mobile phone users compared to non-users |
| ***In vitro* models** | | | | |
| ([Xu et al., 2010](#_ENREF_120)) | Primary cultured neurons | 1800 MHz, pulsed, SAR=2 W/kg, for 24 h | | An increase in the levels of 8-hydroxy-2'-deoxyguanosine (8-OHdG). |
| ([Ding et al., 2018](#_ENREF_30)) | Human semen | 2.45 GHz Wi-Fi, 1.0–2.5 W/kg, for 45 and 90 min | | Reactive oxygen species (ROS) level increased along with longer exposure time. GSH-Px levels decreased, 8-OHdG level increased. |
| ([Agarwal et al., 2009](#_ENREF_2)) | Human sperma-tozoa | Cell phone RFR, in talk mode, for 1 h | | Increase in ROS level, decrease in sperm motility and viability. |
| ([Campisi et al., 2010](#_ENREF_22)) | Rat astroglial cells | 900 MHz (continuous or modulated), electric field 10 V/m, for 5; 10; 20 min | | Increase in ROS levels and DNA fragmentation after exposure to modulated RFR for 20 min. |
| ([De Iuliis et al., 2009](#_ENREF_28)) | Human spermatozoa | 1.8 GHz, SAR=0.4-27.5 W/kg | | Increased amounts of ROS. |
| ([Friedman et al., 2007](#_ENREF_34)) | HeLa membranes | 875 MHz, 200 µW/cm^2^, for 5 and 10 min | | Increased nicotinamide adenine dinucleotide (NADH) oxidase activity. |
| ([Hou et al., 2014](#_ENREF_42)) | Mouse embryonic fibroblasts (NIH/3T3) | 1800-MHz GSM-talk mode RFR, SAR=2 W/kg, intermittent exposure (5 min on/10 min off) for 0.5 to 8 h | | Increased intracellular ROS levels. |
| ([Kahya et al., 2014](#_ENREF_49)) | Cancer cell cultures | 900 MHz RFR, SAR=0.36 W/kg,  for 1 h | | Induced apoptosis effects through oxidative stress, selenium counteracted the effects of RFR exposure. |
| ([Lantow, Lupke, et al., 2006](#_ENREF_61)) | Human blood cells | Continuous wave or GSM signal, SAR=2 W/kg, for 30 or 45 min of continuous or 5 min ON, 5 min OFF | | After continuous or intermittent GSM signal a different ROS production was detected in human monocytes compared to sham. |
| ([Lantow, Schuderer, et al., 2006](#_ENREF_62)) | Human Mono Mac 6 and K562 cells | Continuous wave, GSM speaking only, GSM hearing only, GSM talk, SARs of 0.5, 1.0, 1.5 and 2.0 W/kg. | | The GSM-DTX signal at 2 W/kg produced difference in free radical production compared to sham. |
| ([Liu et al., 2013](#_ENREF_63)) | GC-2 cells | 1800 MHz, SAR=1; 2 W/kg, 5 min ON, 10 min OFF for 24 h | | In the 2 W/kg exposed cultures, the level of ROS was increased. |
| ([Lu et al., 2012](#_ENREF_64)) | Human blood mononuclear cells | 900 MHz, SAR=0.4 W/kg, for 1-8 h | | The increased level of apoptosis induced through the mitochondrial pathway and mediated by activating ROS and caspase-3. |
| ([Marjanovic et al., 2014](#_ENREF_68)) | V79 cells | 1800 MHz, SAR=1.6 W/kg, for 10, 30 and 60 min | | ROS level increased after 10 min of exposure. Decrease in ROS level after 30-min treatment indicating antioxidant defense mechanism activation. |
| ([Naziroglu, Cig, et al., 2012](#_ENREF_78)) | HL-60 cells | 2450 MHz, pulsed, SAR=0.1-2.5 W/kg, for 1; 2; 12 or 24 h | | LPO levels were increased at all exposure times. |
| ([Ni et al., 2013](#_ENREF_80)) | Human lens epithelial cells | 1800 MHz, SAR=2; 3; 4 W/kg | | The ROS and MDA levels were increased. |
| ([Pilla, 2012](#_ENREF_94)) | Neuronal cells and human fibroblasts | 27.12 MHz, pulsed, electric field 41 V/m, 2 min prior to lipopolysaccharide administration or for 15 min, | | Increased level of nitric oxide (NO). |
| ([Sefidbakht et al., 2014](#_ENREF_99)) | HEK293T cells | 940 MHz, SAR=0.09 W/kg, for 15, 30, 45, 60 and 90 min | | ROS generation increased in the 30 min exposed cells. A sharp rise in catalase (CAT) and SOD activity and elevation of GSH during the 45 min exposure. |
| ([Zmyślony et al., 2004](#_ENREF_123)) | Rat lymphocytes | 930 MHz, PD of 500 µW/cm^2^, SAR=1.5 W/kg, for 5 and 15 min | | Intracellular ROS level increased in exposed FeCl_2_ treated cells compared with unexposed FeCl_2_ treated cells. |
| ([Özsobacı et al., 2020](#_ENREF_92)) | Human kidney embryonic cells (HEK293) | 2.45 GHz EMR for 1 hour | | Increased MDA level and decreased SOD activity. |
| ([Özsobacı et al., 2018](#_ENREF_91)) | Human embryonic kidney cells (HEK293) | 2.45 GHz EMR | | MDA levels were increased, SOD and GSH-Px activities were decreased. |
| ([Wang et al., 2020](#_ENREF_119)) | MC3T3-E1 cells | 2.45 GHz Wi-Fi, 100 mW and 500 mW, SAR 0.1671 W/kg and 0.8356 W/kg, for 0-180 min | | Ninety min of Wi-Fi irradiation increased ROS and GSH levels. |
| ([Marjanovic Cermak et al., 2018](#_ENREF_69)) | Human neuroblastoma cells (SH-SY5Y) | 1800 MHz, SAR 1.6 W/kg for 10, 30 and 60 min | | Increased ROS levels were observed for every exposure time, 60 min of exposure caused lipid and protein damage. |
| ([Koohestani et al., 2019](#_ENREF_56)) | Preantral follicles | 1,900 MHz cell phone, SAR 0.77- 0.88 W/kg for 60 min | | SOD, GSH-Px, CAT activity decreased, the TAC level decreased and MDA levels increased. |
| ([Houston et al., 2018](#_ENREF_43)) | Mouse spermatogonial GC1 and spermatocyte GC2 cell lines, cauda epididymal spermatozoa | 1.8 GHz, 0.15 and 1.5 W/kg for 4 hours | | Increased generation of mitochondrial ROS, identifying Complex III of the electron transport chain as the potential source of electrons producing ROS. |
| ([Durdik et al., 2019](#_ENREF_31)) | Umbilical cord blood (UCB) cells | MW pulsed signals from GSM900/UMTS test-mobile phone: 915 MHz or 1947.4 MHz SAR values of 4 and/or 40 mW/kg during 1 h | | Increased ROS level after 1 h of UMTS exposure |
| **Animal models** | | | | |
| ([Lai and Singh, 1997](#_ENREF_60)) | Rat whole body | | 2450 MHz, pulsed, PD=2 mW/cm^2^, SAR=1.2 W/kg | Melatonin or spin-trap compound blocked DNA strand breaks induced by RFR exposure in rat brain cells. |
| ([Khalil et al., 2012](#_ENREF_54)) | Rat whole body | 1800 MHz, electric field 15-20 V/m, for 2 h | | Elevations in the levels of 8-OHdG in urine. |
| ([Kumar et al., 2014](#_ENREF_59)) | Rat whole body | Cell phone 1910.5 MHz RFR, 2 h/day for 60 days  day (6 days a week). | | Increase in LPO, damage in sperm cells, and DNA damage. |
| ([Gürler et al., 2014](#_ENREF_39)) | Rat whole body | 2450 MHz, 3.68 V/m, 1 h/day for 30 days | | Increased 8-OHdG level in both plasma and brain tissue whereas it increased protein oxidation (PO) level only in plasma. |
| ([Yakymenko et al., 2018](#_ENREF_121)) | J. quail embryos | GSM 1800 MHz, 0.32 µW/cm^2^, 48 s – On/12 s - Off, during 5 days before and 14 days through the incubation | | An increase in superoxide anion and in nitrogen oxide generation rate, and oxidative damages of DNA (increased levels of 8-OHdG). |
| ([Mehmet Esref Alkis, Bilgin, et al., 2019](#_ENREF_11)) | Male lab rats | 900 MHz, 1800 MHz or 2100 MHz (0.08 W/kg, 0.05 W/kg, and 0.034 W/kg), 2 h/day for 6 months | | MDA, 8-OHdG, and total oxidant status (TOS) were increased in brain tissues. |
| ([Mehmet Esref Alkis, Akdag, et al., 2019](#_ENREF_10)) | Male lab rats | 900 MHz, 1800 MHz or 2100 MHz (0.003 W/kg, 0.002 W/kg, and 0.001 W/kg for testicles), 2 h/day for 6 months | | TOS, MDA, 8-OHdG and oxidative stress index (OSI) were increased in testes. |
| ([Mehmet E Alkis et al., 2021](#_ENREF_9)) | Male lab rats | 1800 MHz or 2100 MHz (0.62 W/kg or 0.2 W/kg), 2 h/day for 7 months | | Increase in MDA, 8-OHdG, total oxidant status, oxidative stress index, and comet assay tail intensity, while total antioxidant status levels decreased.in liver tissues. |
| ([Akbari et al., 2014](#_ENREF_4)) | Rat whole body | RFR from base transceiver station | | GSH-Px, SOD, and CAT activity decreased and level of MDA increased. Vitamin C reduced the effect. |
| ([Al-Damegh, 2012](#_ENREF_8)) | Rat whole body | Cell phone RFR, 15, 30, or 60 min/day for 2 weeks | | Levels of conjugated dienes, LPO and CAT activities in serum and testicular tissue increased, the total serum and testicular tissue GSH and GSH-Px levels decreased. |
| ([Avci et al., 2012](#_ENREF_13)) | Rat whole body | 1800 MHz, SAR=0.4 W/kg, 1 h/day for 3 weeks | | An increased level of PO in brain tissue and an increase in serum NO. |
| ([Ayata et al., 2004](#_ENREF_14)) | Rat whole body | 900 MHz, 30 min/day for 10 days | | MDA and hydroxyproline levels and activities of CAT and GSH-Px were increased, and SOD activity was decreased in skin. Melatonin treatment reversed effect. |
| ([Aynali et al., 2013](#_ENREF_15)) | Rat whole body | 2450 MHz, pulsed, SAR=0.143 W/kg, 60 min/day for 30 days | | LPO was increased, an administration of melatonin prevented this effect. |
| ([Balci et al., 2007](#_ENREF_17)) | Rat whole body | “Standardized daily dose” of cell phone RFR for 4 weeks | | In corneal tissue, MDA level and CAT activity increased, whereas SOD activity was decreased. In the lens tissues, the MDA level was increased. |
| ([Bilgici et al., 2013](#_ENREF_18)) | Rat whole body | 850-950 MHz, SAR=1.08 W/kg, 1 h/day for 3 weeks | | The serum NO levels and levels of MDA and the PO in brain were increased. An administration of garlic extract diminished these effects. |
| ([Bodera et al., 2013](#_ENREF_19)) | Rat whole body | 1800 MHz, GSM, for 15 min | | Reduced antioxidant capacity both in healthy animals and in those with paw inflammation. |
| ([A Burlaka et al., 2013](#_ENREF_21)) | Quail embryo *in ovo* | GSM 900 MHz, power density (PD) of 0.25 µW/cm^2^, SAR=3 µW/kg, 48 sec ON - 12 sec OFF, for 158-360 h | | Overproduction of superoxide and NO, increased levels of thiobarbituric acid reactive substances (TBARS) and 8-OH-dG, decreased SOD and CAT activities. |
| ([Anatoly Burlaka et al., 2014](#_ENREF_20)) | Male rat whole body | Pulsed and continuous MW in the doses equivalent to the maximal permitted energy load for the staffs of the radar stations | | Increased rates of superoxide production, formation of the iron-nitrosyl complexes, and decreased activity of NADH-ubiquinone oxidoreductase complex in liver, cardiac and aorta tissues 28 days after the exposure. |
| ([Çenesi̇z et al., 2011](#_ENREF_23)) | Guinea pig whole body | 900; 1800 MHz RFR from base station antennas, 4 h/day for 20 days | | Difference in guinea pigs subjected to 900 and 1800 MHz for plasma oxidant status levels. NO level changed in 900 MHz subjected guinea pigs, as compared to the control. |
| ([Çetin et al., 2014](#_ENREF_24)) | Pregnant rats and offspring | 900; 1800 MHz RFR, 1 h/day during pregnancy and neonatal development | | Brain and liver GSH-Px activities, selenium concentrations in the brain, and liver vitamin A and β-carotene concentrations decreased in offspring. |
| ([Dasdag et al., 2009](#_ENREF_26)) | Head of rats | 900 MHz, 2h/day for 10 months | | The total antioxidant capacity and CAT activity in brains were higher than that in the sham group. |
| ([Dasdag et al., 2012](#_ENREF_25)) | Head of rats | 900 MHz, cell-phones-like, 2 h/day for 10 months | | Protein carbonyl level was higher in the brain of exposed rats. |
| ([Dasdag et al., 2008](#_ENREF_27)) | Rat whole body | 900 MHz, PD of 78 µW/cm^2^, 2 h/days for 10 months. | | Increased levels of MDA and total oxidative status in liver tissue. |
| ([Deshmukh et al., 2013](#_ENREF_29)) | Rat whole body | 900 MHz, 2 h/day, 5 days a week for 30 days | | The levels of LPO and PO were increased. |
| ([Esmekaya et al., 2011](#_ENREF_33)) | Rat whole body | 900 MHz, pulsed, modulated, SAR=1.2 W/kg, 20 min/day for 3 weeks | | The increased level of MDA and NOx, and decreased levels of GSH in liver, lung, testis and heart tissues. |
| ([Furtado-Filho et al., 2014](#_ENREF_35)) | Rat whole body | 950 MHz, SAR=0.01-0.88 W/kg, 30 min/day for 21 days during pregnancy (or additionally 6 or 15 days of postnatal period) | | Neonatal rats exposed in utero had decreased levels of CAT and lower LPO, and genotoxic effect. |
| ([Guney et al., 2007](#_ENREF_38)) | Rat whole body | 900 MHz, 30 min/day for 30 days | | Endometrial levels of NO and MDA increased, endometrial SOD, CAT and GSH-Px activities were decreased. Vitamin E and C treatment prevented these effects. |
| ([Ilhan et al., 2004](#_ENREF_44)) | Rat whole body | 900 MHz, from cell phone,1 h/day for 7 days | | Increase in MDA, NO levels, and xanthine oxidase (XO) activity, decrease in SOD and GSH-Px activities in brain. These effects were prevented by Ginkgo biloba extract treatment. |
| ([Jelodar, Akbari, et al., 2013](#_ENREF_46)) | Rat whole body | 900 MHz, PD of 680 µW/cm^2^, 4 h/day for 45 days, | | The concentration of MDA was increased and activities of SOD, GSH-Px and CAT were decreased in rat eyes. An administration of vitamin C prevented these effects. |
| ([Jelodar, Nazifi, et al., 2013](#_ENREF_47)) | Rat whole body | 900 MHz, daily for 45 days | | Increased level of MDA and decreased antioxidant enzymes activity in rat testis. |
| ([Jing et al., 2012](#_ENREF_48)) | Rat whole body | Cell phone RFR, SAR=0.9 W/kg, 3 x 10; 30 or 60 min for 20 days during gestation | | After 30 and 60 min the level of MDA was increased, the activities of SOD and GSH-Px were decreased. |
| ([Kerman and Senol, 2012](#_ENREF_51)) | Rat whole body | 900 MHz, 30 min/day for 10 days | | Tissue MDA levels were increased, SOD, CAT and GSH-Px activities were reduced. Melatonin treatment reversed these effects. |
| ([Kesari et al., 2011](#_ENREF_52)) | Rat whole body | 900 MHz, pulsed, SAR=0.9 W/kg, 2 h/day for 45 days | | Increase in the level of ROS, decrease in the activities of SOD and GSH-Px, and in the level of pineal melatonin. |
| ([Kesari et al., 2014](#_ENREF_53)) | Rat whole body | 2115 MHz, SAR=0.26 W/kg, 2 h/day for 60 days | | The level of ROS, DNA damage and the apoptosis rate were increased. |
| ([Koc et al., 2013](#_ENREF_55)) | Male rat whole body | Cell phone RFR at calling or stand-by | | Oxidative stress detected at both calling and stand-by exposures. |
| ([Koylu et al., 2006](#_ENREF_57)) | Rat whole body | 900 MHz | | The levels of LPO in the brain cortex and hippocampus increased. These levels in the hippocampus were decreased by melatonin administration. |
| ([Koyu et al., 2009](#_ENREF_58)) | Rat whole body | 900 MHz | | The activities of XO, CAT and level of LPO increased in liver. XO, CAT activities and LPO levels were decreased by caffeic acid phenethyl ester (CAPE) administration. |
| ([Luo et al., 2014](#_ENREF_65)) | Rat whole body | 900 MHz imitated cell phone RFR, 4 h/day for 12 days | | Contents of liver MDA and Nrf2 protein increased, contents of liver SOD and GSH decreased. |
| ([Mailankot et al., 2009](#_ENREF_66)) | Rat whole body | 900/1800 MHz, GSM, 1 h/day for 28 days | | Increase in LPO and decreased GSH content in the testis and epididymis. |
| ([Manta et al., 2013](#_ENREF_67)) | Drosophila whole body | 1880-1900 MHz, DECT modulation, SAR=0.009 W/kg, for 0.5-96 h | | Increase in ROS levels in male and female bodies, a quick response in ROS increase in ovaries. |
| ([Marzook et al., 2014](#_ENREF_70)) | Rat whole body | 900 MHz from cellular tower, 24 h/day for 8 weeks | | SOD and CAT activities were reduced in blood, sesame oil reversed the effect |
| ([Meena et al., 2013](#_ENREF_72)) | Rat whole body | 2450 MHz, PD of 210 µW/cm^2^, SAR=0.14 W/kg, 2 h/day for 45 days | | Increased level of MDA and ROS in testis. Melatonin prevented oxidative stress. |
| ([Megha et al., 2012](#_ENREF_73)) | Rat whole body | 900; 1800 MHz, PD of 170 µW/cm^2^, SAR=0.6 mW/kg, 2 h/day, 5 days/week for 30 days | | The levels of the LPO and PO were increased; the level of GSH was decreased. |
| ([Meral et al., 2007](#_ENREF_74)) | Guinea pig whole body | 890-915 MHz, from cell phone, SAR=0.95 w/kg, 12 h/day for 30 days (11 h 45 min stand-by and 15 min spiking mode) | | MDA level increased, GSH level and CAT activity were decreased in the brain. MDA, vitamins A, D_3_ and E levels, and CAT enzyme activity increased, and GSH level was decreased in the blood. |
| ([Motawi et al., 2014](#_ENREF_75)) | Rat whole body | Test cellphone RFR, SAR=1.13 W/kg, 2 h/day for 60 days | | Increments in conjugated dienes, protein carbonyls, total oxidant status, and oxidative stress index along with a reduction of total antioxidant capacity levels. |
| ([Naziroglu and Gumral, 2009](#_ENREF_79)) | Rat whole body | 2450 MHz, 60 min/day for 28 days | | Decrease of the cortex brain vitamin A, vitamin C and vitamin E levels. |
| ([Naziroglu, Celik, et al., 2012](#_ENREF_77)) | Rat whole body | 2450 MHz, 60 min/day for 30 days | | LPO, cell viability and cytosolic Ca^2+^ values in dorsal root ganglion neurons were increased. |
| ([Oksay et al., 2014](#_ENREF_81)) | Rat whole body | 2450 MHz, pulsed, PD of 0.1 µW/cm^2^, SAR=0.1 W/kg, 1 h/day for 30 days | | LPO was higher in exposed animals. Melatonin treatment reversed the effect. |
| ([Oktem et al., 2005](#_ENREF_82)) | Rat whole body | 900 MHz, 30 min/day for 10 days | | Renal tissue MDA level increased, SOD, CAT, and GSH-Px activities were reduced. Melatonin treatment reversed these effects. |
| ([Oral et al., 2006](#_ENREF_83)) | Rat whole body | 900 MHz, 30 min/day for 30 days | | Increased MDA levels and apoptosis in endometrial tissue. Treatment with vitamins E and C diminished these changes. |
| ([Ozguner, Altinbas, et al., 2005](#_ENREF_85)) | Rat whole body | 900 MHz, 30 min/day for 10 days | | Heart tissue MDA and NO levels increased, SOD, CAT and GSH-Px activities were reduced. CAPE treatment reversed these effects. |
| ([Ozguner et al., 2006](#_ENREF_86)) | Rat whole body | 900 MHz, from cell phone | | Retinal levels of NO and MDA increased, SOD, GSH-Px and CAT activities were decreased. Melatonin and CAPE treatment prevented effects. |
| ([Ozguner, Oktem, et al., 2005](#_ENREF_87)) | Rat whole body | 900 MHz | | Renal tissue MDA and NO levels increased, the activities of SOD, CAT, and GSH-Px were reduced. CAPE treatment reversed these effects. |
| ([E. Ozgur et al., 2010](#_ENREF_88)) | Guinea pig whole body | 1800 MHz, GSM, SAR=0.38 W/kg, 10 or 20 min/day for 7 days | | Increases in MDA and total NO(x) levels and decreases in activities of SOD, myeloperoxidase and GSH-Px in liver. Extent of oxidative damage was proportional to the duration of exposure. |
| ([Elcin Ozgur et al., 2013](#_ENREF_89)) | Rabbit whole body | 1800 MHz, pulsed, 15 min/day for 7 days in pregnant animals, for 7 or 15 days in infants | | The amount of LPO was increased in the prenatal exposure group. |
| ([Özorak et al., 2013](#_ENREF_90)) | Rat whole body | 900; 1800; 2450 MHz, pulsed, PD of 12 µW/cm^2^.SAR=0.18; 1.2 W/kg, 60 min/day during gestation and 6 weeks following delivery | | At the age of six weeks, an increased LPO in the kidney and testis, and decreased level of GSH and total antioxidant status. |
| ([Qin et al., 2014](#_ENREF_95)) | Male mouse whole body | 1800 MHz, 208 µW/cm^2^, 30 or 120 min/d for 30 days | | Decreased activities of CAT and GSH-Px and increased level of MDA in cerebrum. Nano-selenium decreased MDA level, and increased GSH-Px and CAT activities. |
| ([Ragy, 2014](#_ENREF_96)) | Rat whole body | Cell phone 900 MHz RFR, 1 h/d for 60 days | | Increase in MDA levels and decrease total antioxidant capacity levels in brain, liver and kidneys tissues. These alterations were corrected by withdrawal of RFR exposure during 30 days. |
| ([Saikhedkar et al., 2014](#_ENREF_97)) | Rat whole body | Cell phone 900 MHz RFR, 4 h/d for 15 days | | A significant change in level of antioxidant enzymes and non-enzymatic antioxidants, and an increase in LPO. |
| ([Shahin et al., 2013](#_ENREF_101)) | Mouse whole body | 2450 MHz, PD of 33.5 µW/cm^2^, SAR=23 mW/kg, 2 h/day for 45 days | | An increase in ROS, decrease in NO and antioxidant enzymes activities. |
| ([Sokolovic et al., 2008](#_ENREF_107)) | Rat whole body | RFR from cell phone, SAR=0.043–0.135 W/kg, for 20, 40 and 60 days | | An increase in the brain tissue MDA and carbonyl group concentration. Decreased activity of CAT and increased activity of xanthine oxidase (XO). Melatonin treatment prevented the effects. |
| ([Sokolovic et al., 2013](#_ENREF_108)) | Rat whole body | 900 MHz, SAR=0,043-0.135 W/kg, 4 h/day for 29; 40 or 60 days, | | The level of LPO and PO, activities of CAT, XO, number of apoptotic cells were increased in thymus tissue. An administration of melatonin prevented these effects. |
| ([Dasdag et al., 2004](#_ENREF_109)) | Rat whole body | Cell phone RFR, SAR=0.52 W/kg, 20 min/day for 1 month | | MDA concentration was increased in brains. |
| ([Tkalec et al., 2007](#_ENREF_110)) | Plant Lemna minor (duckweed) | 400 and 900 MHz, 10, 23, 41 and 120 V/m, for 2 or 4 h | | LPO and H_2_O_2_ content increased: CAT activity increased, pyrogallol peroxidase decreased. |
| ([Tkalec et al., 2013](#_ENREF_111)) | Earthworm whole body | 900 MHz, PD of 30-3800 µW/cm^2^, SAR=0.13-9.33 mW/kg, for 2 h | | The protein carbonyl content was increased in all exposures above 30 µWc/m^2^. The level of MDA was increased at 140 µW/cm^2^. |
| ([Tök et al., 2014](#_ENREF_112)) | Rat whole body | 2450 MHz, Wi-Fi RFR, 60 min/day for 30 days | | Decreased GSH-Px activity. GSH-Px activity and GSH values increased after melatonin treatment. |
| ([Tomruk et al., 2010](#_ENREF_113)) | Rabbit whole body | 1800 MHz, GSM-like signal, 15 min/day for a week | | Increase of MDA and ferrous oxidation in xylenol orange levels. |
| ([Tsybulin et al., 2012](#_ENREF_115)) | Quail embryo *in ovo* | 900 MHz, from cell phone, GSM, PD of 0.024-0.21 µW/cm^2^, intermittent for 14 days | | Increased level of TBARS in brains and livers of hatchlings. |
| ([Turker et al., 2011](#_ENREF_117)) | Rat partial body | 2450 MHz, pulsed, SAR=0.1 W/kg, 1 h/day for 28 days | | The increased level of LPO, the decreased concentrations of vitamin A, vitamin C, and vitamin E. There was a protective effect of selenium and L-carnitine. |
| ([Türedi et al., 2014](#_ENREF_116)) | Pregnant rat whole body | 900 MHz, 13.7 V/m, 50 µW/cm^2^, 1 h/day for 13-21 days of pregnancy | | MDA, SOD and CAT values increased, GSH values decreased in exposed pups. |
| ([Yurekli et al., 2006](#_ENREF_122)) | Rat whole body | 945 MHz, GSM, PD of 367 µW/cm^2^, SAR=11.3 mW/kg | | MDA level and SOD activity increased, GSH concentration was decreased. |
| ([Ismaiil et al., 2019](#_ENREF_45)) | Male lab rats | GSM 900 MHz mobile phone, 24 h/day for 28 days | | Increased hepatic levels of MDA and Nrf-2, activities of SOD and catalase. |
| ([Ahmed et al., 2017](#_ENREF_3)) | Lab rats | 900 MHz modulated at 217 Hz, 0.02 mW/cm2, SAR 1.245 W/kg for 3 months | | EMR exposure resulted in oxidative stress in the hippocampus and striatum. |
| ([Gautam et al., 2019](#_ENREF_37)) | Male lab rats | 3G mobile phone, 2 h/day for 45 days | | Increase in ROS and lipid peroxidation levels with decrease in sperm count, alterations in sperm tail morphology. |
| ([Bahreyni Toossi et al., 2018](#_ENREF_16)) | Pregnant female lab mice | Mobile phone (900–1800 MHz), 2 h/day for 20 days during pregnancy | | MDA levels were increased, while total thiol groups (TTG), SOD, and CAT were decreased in the tissues of dams and their offspring. |
| ([Oyewopo et al., 2017](#_ENREF_84)) | Lab rats | Radiation of cell phone for 28 days. | | Increased sera levels of MDA and decreased SOD activity. |
| ([Ertilav et al., 2018](#_ENREF_32)) | Lab rats | 900 MHz or 1800 MHz, 60 min/ 5 days of the week during one year | | Increases in intracellular free calcium influx (Ca2+), ROS production, mitochondrial membrane depolarization, apoptosis, and caspase 3 and 9 activities. |
| ([Zosangzuali et al., 2021](#_ENREF_124)) | Lab mice | Mobile phone base station 1800-MHz, for 12 hr or 24 hr per day for 45 days | | Increase in MDA levels in brain. |
| ([S. Sharma and Shukla, 2020](#_ENREF_103)) | Lab rats | 900 MHz, 1 h, 2 h, or 4 h for 90 days | | Increased level of MDA with depleted levels of SOD, CAT and redox enzymes (GSH, GPX, GR, GST, G-6PDH). |
| ([Kamali et al., 2018](#_ENREF_50)) | Lab rats | Wi-Fi 2.45 GHz radiation, 24 h/day for 10 weeks | | Decreased total antioxidant capacity of plasma and the activities of CAT, GSH-Px, and SOD. The GST activity was increased. |
| ([Asl et al., 2020](#_ENREF_12)) | Lab rats | 915 MHz (mobile phone) and 2450 MHz (Wi-Fi) for one month | | Elevation of protein carbonylation (PC), nitric oxide (NO) and MDA, and reduction in GSH, GSH-Px, SOD and CAT in brain. |
| ([Saygin et al., 2018](#_ENREF_98)) | Female lab rats | 2.45 GHz Wi-Fi, 1 h/day for 30 days | | Total oxidant status (TOS) and oxidative stress index (OSI) levels increased in ovarian tissues. |
| ([Masoumi et al., 2018](#_ENREF_71)) | Male lab rats | 2.45 GHz Wi-Fi, 4 h/day for 45 days | | An increase in lipid peroxidation and a decrease in GSH level, SOD, and GPx activities of the pancreas, hyperglycemia. |
| ([Usman et al., 2020](#_ENREF_118)) | Lab rats | Multiple transceiver mobile phone | | A decrease in the levels of glutathione peroxidase, SOD, and MDA in the serum, heart and the brain. CAN decreased in the brain. Nitric oxide activities increased in the brain and heart. |
| ([Akefe et al., 2019](#_ENREF_6)) | Lab mice | 900 MHz, 1 y/day for 28 days | | Reduced activities of brain antioxidant enzymes, increased lipoperoxidation, and impairment in learning and memory. |
| ([Hancı et al., 2018](#_ENREF_41)) | Male lab rats | Continuous 900-MHz, 1 h/day during adolescence | | Changes in oxidative stress biomarkers and in morphology of the rat testis. |
| ([Topsakal et al., 2017](#_ENREF_114)) | Male lab rats | 2.45 GHz Wi-Fi for 30 days | | Tissue MDA, total oxidant status and oxidative stress index were increased, whereas total antioxidant status decreased. |
| ([Shedid et al., 2019](#_ENREF_105)) | Male lab rats | 950 MHz, 1 mW/cm^2^, SAR 0.238 and 0.372 W/kg, 1 hour thrice a week for seven weeks | | Increase of MDA and NO, decrease in the activity of SOD, CAT and GSH-Px in liver and brain. |
| ([Pandey and Giri, 2018](#_ENREF_93)) | Male lab mice | GSM 900 MHz for 3 h twice/day for 35 days | | Excess free radical generation, extensive DNA damage in germ cells. |
| ([A. Sharma et al., 2020](#_ENREF_102)) | Male lab rats | 1800 MHz, 0.433 W/kg, 4 h/5 days/week for 90 days | | Alteration in GSH cycle regulating enzymes such as GSH reductase, GSH-Px, GSH transferase, and increase in the DNA damage in brain neurons. |
| **Plant models** | | | | |
| ([V. P. Sharma et al., 2009](#_ENREF_104)) | Plant (mung bean) | 900 MHz, from cell phone, PD of 8.55 µW/cm^2^; for 0.5; 1; 2, and 4 h | | Increased level of MDA, H_2_O_2_ accumulation and root oxidizability, upregulation in the activities of SOD, CAT, ascorbate peroxidases, guaiacol peroxidases, and GSH reductases in roots. |
| ([Singh et al., 2012](#_ENREF_106)) | Plant (mung bean) | 900 MHz, from cell phone | | The increased level of MDA, hydrogen peroxide, and proline content in hypocotyls. |

*All effects were statistically significant (at least p<0.05) as compared to control or sham exposed groups.

**References**

Abu Khadra KM, Khalil AM, Abu Samak M. et al. Evaluation of selected biochemical parameters in the saliva of young males using mobile phones. Electromagn Biol Med. 2015;34:72-6.

Agarwal A, Desai NR, Makker K, et al. Effects of radiofrequency electromagnetic waves (RF-EMW) from cellular phones on human ejaculated semen: an in vitro pilot study. Fertil Steril 2009;92:1318-25.

Ahmed NA, Radwan NM, Aboul Ezz HS, et al. The antioxidant effect of Green Tea Mega EGCG against electromagnetic radiation-induced oxidative stress in the hippocampus and striatum of rats. Electromagn Biol Med. 2017;36:63-73.

Akbari A, Jelodar G, Nazifi, S. Vitamin C protects rat cerebellum and encephalon from oxidative stress following exposure to Radiofrequency wave generated by BTS antenna mobile. Toxicol MechMethods. 2014;24:347-52.

Akdag M, Dasdag S, Canturk F, et al. Exposure to non-ionizing electromagnetic fields emitted from mobile phones induced DNA damage in human ear canal hair follicle cells. Electromagn Biol Med. 2018;37:66-75.

Akefe IO, Yusuf IL, Adegoke VA. C-glycosyl flavonoid orientin alleviates learning and memory impairment by radiofrequency electromagnetic radiation in mice via improving antioxidant defence mechanism. Asian Pac J Trop Biomed. 2019;9:518-23.

Akkam Y, Al-Taani AA, Ayasreh S, et al. Correlation of blood oxidative stress parameters to indoor radiofrequency radiation: A cross sectional study in Jordan. Int J Environ Res Public Health 2020;17:4673. doi: 10.3390/ijerph17134673.

Al-Damegh MA. Rat testicular impairment induced by electromagnetic radiation from a conventional cellular telephone and the protective effects of the antioxidants vitamins C and E. Clinics 2012;67:785-792.

Alkis ME, Akdag MZ, Dasdag S. Effects of Low‐Intensity Microwave Radiation on Oxidant‐Antioxidant Parameters and DNA Damage in the Liver of Rats. Bioelectromagnetics. 2021;42:76-85.

Alkis ME, Akdag MZ, Dasdag S, et al. Single-strand DNA breaks and oxidative changes in rat testes exposed to radiofrequency radiation emitted from cellular phones. Biotechnol. Biotechnol. Equip. 2019;33:1733-40.

Alkis ME, Bilgin HM, Akpolat V, et al. Effect of 900-, 1800-, and 2100-MHz radiofrequency radiation on DNA and oxidative stress in brain. Electromagn Biol Med. 2019;38:32-47.

Asl JF, Goudarzi M, Shoghi H. The radio-protective effect of rosmarinic acid against mobile phone and Wi-Fi radiation-induced oxidative stress in the brains of rats. Pharmacol. Rep. 2020;72:857-866.

Avci B, Akar A, Bilgici B, et al. Oxidative stress induced by 1.8 GHz radio frequency electromagnetic radiation and effects of garlic extract in rats. Int J Radiat Biol 2012;88:799-805.

Ayata A, Mollaoglu H, Yilmaz HR, et al. Oxidative stress-mediated skin damage in an experimental mobile phone model can be prevented by melatonin. J Dermatol. 2004;31:878-83.

Aynali G, Naziroglu M, Celik O, et al. Modulation of wireless (2.45 GHz)-induced oxidative toxicity in laryngotracheal mucosa of rat by melatonin. Eur Arch Otorhinolaryngol. 2013;270:1695-1700.

Bahreyni Toossi MH, Sadeghnia HR, Feyzabadi MMM, et al. Exposure to mobile phone (900–1800 MHz) during pregnancy: tissue oxidative stress after childbirth*.* J. Matern. Fetal Neonatal Med. 2018;31:1298-1303.

Bektas H, Dasdag S, Bektas MS. Comparison of effects of 2.4 GHz Wi-Fi and mobile phone exposure on human placenta and cord blood. Biotechnol. Biotechnol Equip. 2020;34:154-162.

Balci M, Devrim E, Durak I. Effects of mobile phones on oxidant/antioxidant balance in cornea and lens of rats. Curr Eye Res. 2007;32:21-25.

Bilgici B, Akar A, Avci B, et al. Effect of 900 MHz radiofrequency radiation on oxidative stress in rat brain and serum. Electromagn Biol Med. 2013;32:20-29.

Bodera P, Stankiewicz W, Zawada K, et al. Changes in antioxidant capacity of blood due to mutual action of electromagnetic field (1800 MHz) and opioid drug (tramadol) in animal model of persistent inflammatory state. Pharmacol Rep. 2013;65:421-8.

Burlaka A, Selyuk M, Gafurov M, et al. Changes in mitochondrial functioning with electromagnetic radiation of ultra high frequency as revealed by electron paramagnetic resonance methods. Int J Radiat Biol. 2014;90:357-62.

Burlaka A, Tsybulin O, Sidorik, E, et al. Overproduction of free radical species in embryonic cells exposed to low intensity radiofrequency radiation. Exp Oncol. 2013; 35:219-25.

Campisi A, Gulino M, Acquaviva R, et al. Reactive oxygen species levels and DNA fragmentation on astrocytes in primary culture after acute exposure to low intensity microwave electromagnetic field. Neurosci Lett. 2010;473:52-5.

Çenesi̇z M, Ataki̇şi̇ O, Akar A, et al. Effects of 900 and 1800 MHz electromagnetic field application on electrocardiogram, nitric oxide, total antioxidant capacity, total oxidant capacity, total protein, albumin and globulin levels in guinea pigs. Kafkas Üniv Vet Fak Derg. 2011;17:357-62.

Çetin H, Naziroglu M, Çelik Ö, et al. Liver antioxidant stores protect the brain from electromagnetic radiation (900 and 1800 MHz)-induced oxidative stress in rats during pregnancy and the development of offspring. J Matern Fetal Neonatal Med. 2014;27:1915-21.

Dasdag S, Akdag MZ, Kizil G, et al. Effect of 900 MHz radio frequency radiation on beta amyloid protein, protein carbonyl, and malondialdehyde in the brain. Electromagn Biol Med. 2012;31:67-74.

Dasdag S, Akdag MZ, Ulukaya E, et al. Effect of mobile phone exposure on apoptotic glial cells and status of oxidative stress in rat brain. Electromagn Biol Med. 2009;28:342-54.

Dasdag S, Bilgin H, Akdag MZ, et al. Effect of long term mobile phone exposure on oxidative-antioxidative processes and nitric oxide in rats. Biotechnol Biotechnol Equip. 2008;22:992-7.

Dasdag S, Akdag MZ, Feyzan A, et al. Does 900 MHZ GSM mobile phone exposure affect rat brain? Electromagn Biol Med. 2004;23:201-14.

De Iuliis GN, Newey RJ, King BV, et al. Mobile phone radiation induces reactive oxygen species production and DNA damage in human spermatozoa in vitro. PLoS One 2009;4:e6446. doi: 10.1371/journal.pone.0006446.

Deshmukh PS, Banerjee BD, Abegaonkar MP, et al. Effect of low level microwave radiation exposure on cognitive function and oxidative stress in rats. Indian J Biochem Biophys 2013;50:114-19.

Ding SS, Sun P, Zhang Z, et al. Moderate dose of trolox preventing the deleterious effects of Wi-Fi radiation on spermatozoa in vitro through reduction of oxidative stress damage. Chinese Med J. (Engl) 2018;131:402-12.

Durdik M, Kosik P, Markova E, et al. Microwaves from mobile phone induce reactive oxygen species but not DNA damage, preleukemic fusion genes and apoptosis in hematopoietic stem/progenitor cells. Scientific Reports 2019;9:16182. doi:[10.1038/s41598-019-52389-x](https://www.nature.com/articles/s41598-019-52389-x)

Ertilav K, Uslusoy F, Ataizi S, et al. Long term exposure to cell phone frequencies (900 and 1800 MHz) induces apoptosis, mitochondrial oxidative stress and TRPV1 channel activation in the hippocampus and dorsal root ganglion of rats. Metab Brain Dis. 2018;33:753-63.

Esmekaya MA, Ozer C, Seyhan, N. 900 MHz pulse-modulated radiofrequency radiation induces oxidative stress on heart, lung, testis and liver tissues. Gen Physiol Biophys. 2011;30:84-9.

Friedman J, Kraus S, Hauptman Y, et al. Mechanism of short-term ERK activation by electromagnetic fields at mobile phone frequencies. Biochem J. 405:559-68.

Furtado-Filho OV, Borba, JB, Dallegrave, A, et al. Effect of 950 MHz UHF electromagnetic radiation on biomarkers of oxidative damage, metabolism of UFA and antioxidants in the livers of young rats of different ages. Int J Radiat Biol. 2014;90:159-68.

Garaj-Vrhovac V, Gajski G, Pažanin S, et al. Assessment of cytogenetic damage and oxidative stress in personnel occupationally exposed to the pulsed microwave radiation of marine radar equipment. Int J Hyg Environ Health. 2011;214:59-65.

Gautam R, Singh KV, Nirala J, et al. Oxidative stress‐mediated alterations on sperm parameters in male Wistar rats exposed to 3G mobile phone radiation. Andrologia 2019;51:e13201. doi: 10.1111/and.13201.

Guney M, Ozguner F, Oral B, et al. 900 MHz radiofrequency-induced histopathologic changes and oxidative stress in rat endometrium: protection by vitamins E and C. Toxicol Ind Health. 2007;23:411-20.

Gürler HŞ, Bilgici B, Akar AK, et al. Increased DNA oxidation (8-OHdG) and protein oxidation (AOPP) by low level electromagnetic field (2.45 GHz) in rat brain and protective effect of garlic. Int J Radiat Biol. 2014;90:892-6.

Hamzany Y, Feinmesser R, Shpitzer T, et al. Is human saliva an indicator of the adverse health effects of using mobile phones? Antioxid Redox Signal. 2013;18:622-627.

Hancı H, Kerimoğlu G, Mercantepe T, et al. Changes in testicular morphology and oxidative stress biomarkers in 60-day-old Sprague Dawley rats following exposure to continuous 900-MHz electromagnetic field for 1h a day throughout adolescence. Reprod Toxicol. 2018;81:71-8.

Hou Q, Wang M, Wu S, et al. Oxidative changes and apoptosis induced by 1800-MHz electromagnetic radiation in NIH/3T3 cells. Electromagn Biol Med. 2015;34:85-92.

Houston BJ, Nixon B, King BV, et al. Probing the origins of 1,800 MHz radio frequency electromagnetic radiation induced damage in mouse immortalized germ cells and spermatozoa in vitro. Front Public Health 2018;6:270. doi: 10.3389/fpubh.2018.00270.

Ilhan A, Gurel A, Armutcu F, et al. Ginkgo biloba prevents mobile phone-induced oxidative stress in rat brain. Clin Chim Acta. 2004;340:153-62.

Ismaiil LA, Joumaa WH, Moustafa ME. The impact of exposure of diabetic rats to 900 MHz electromagnetic radiation emitted from mobile phone antenna on hepatic oxidative stress. Electromagn Biol Med. 2019;38:287-96.

Jelodar, G., Akbari, A., Nazifi, S. (2013). The prophylactic effect of vitamin C on oxidative stress indexes in rat eyes following exposure to radiofrequency wave generated by a BTS antenna model. Int J Radiat Biol. 2013;89:128-31.

Jelodar G, Nazifi S, Akbari A. The prophylactic effect of vitamin C on induced oxidative stress in rat testis following exposure to 900 MHz radio frequency wave generated by a BTS antenna model. *Electromagn Biol Med 32*(3):409-416.

Jing J, Yuhua Z, Xiao-qian Y, et al. The influence of microwave radiation from cellular phone on fetal rat brain. Electromagn Biol Med. 2012;31:57-66.

Kahya MC, Nazıroğlu M, Çiğ B. Selenium reduces mobile phone (900 MHz)-induced oxidative stress, mitochondrial function, and apoptosis in breast cancer cells. Biol Trace Elem Res. 2014;160:285-93.

Kamali K, Taravati A, Sayyadi S, et al. Evidence of oxidative stress after continuous exposure to Wi-Fi radiation in rat model. Environ Sci Pollut Res Int. 2018;25:35396-35403.

Kerman M, Senol N. Oxidative stress in hippocampus induced by 900 MHz electromagnetic field emitting mobile phone: Protection by melatonin Biomed Res. 2012;23:147-51.

Kesari KK, Kumar S, Behari, J. 900-MHz microwave radiation promotes oxidation in rat brain. Electromagn Biol Med. 2011;30:219-34.

Kesari KK, Meena R, Nirala J, et al. Effect of 3G cell phone exposure with computer controlled 2-D stepper motor on non-thermal activation of the hsp27/p38MAPK stress pathway in rat brain. Cell Biochem Biophys. 2014;68:347-58.

Khalil AM, Gagaa MH, Alshamali AM. 8-Oxo-7, 8-dihydro-2'-deoxyguanosine as a biomarker of DNA damage by mobile phone radiation. Hum Exp Toxicol. 2012;31:734-40.

Koc A, Unal D, Cimentepe E. The effects of antioxidants on testicular apoptosis and oxidative stress produced by cell phones. Turk J Med Sci. 2013;43:131-7.

Koohestani NV, Zavareh S, Lashkarbolouki T, et al. Exposure to cell phone induce oxidative stress in mice preantral follicles during in vitro cultivation: An experimental study. Int J Reprod BioMed. 2019;17:637-46.

Koylu H, Mollaoglu H, Ozguner F, et al. Melatonin modulates 900 Mhz microwave-induced lipid peroxidation changes in rat brain. Toxicol Ind Health. 2006;22:211-16.

Koyu A, Ozguner F, Yilmaz H, et al. The protective effect of caffeic acid phenethyl ester (CAPE) on oxidative stress in rat liver exposed to the 900 MHz electromagnetic field. Toxicol Ind Health. 2009;25:429-34.

Kumar S, Nirala JP, Behari J, et al. Effect of electromagnetic irradiation produced by 3G mobile phone on male rat reproductive system in a simulated scenario. Indian J Exp. Biol. 2014;52:890-7.

Lai H, Singh N P. Melatonin and a spin-trap compound block radiofrequency electromagnetic radiation-induced DNA strand breaks in rat brain cells. Bioelectromagnetics. 1997;18:446-54.

Lantow M, Lupke M, Frahm J, et al. ROS release and Hsp70 expression after exposure to 1,800 MHz radiofrequency electromagnetic fields in primary human monocytes and lymphocytes. Radiat Environ Biophys. 2006;45:55-62.

Lantow M, Schuderer J, Hartwig, C, et al. Free radical release and HSP70 expression in two human immune-relevant cell lines after exposure to 1800 MHz radiofrequency radiation. Radiat Res. 2006;165:88-94.

Liu C, Duan W, Xu S, et al. Exposure to 1800 MHz radiofrequency electromagnetic radiation induces oxidative DNA base damage in a mouse spermatocyte-derived cell line. Toxicol Lett. 2013;218:2-9.

Lu YS, Huang BT, Huang YX. Reactive oxygen species formation and apoptosis in human peripheral blood mononuclear cell induced by 900 MHz mobile phone radiation. Oxid Med Cell Longev 2012:740280. doi: 10.1155/2012/740280.

Luo YP, Ma HR, Chen JW, et al. Effect of American Ginseng Capsule on the liver oxidative injury and the Nrf2 protein expression in rats exposed by electromagnetic radiation of frequency of cell phone. Zhongguo Zhong Xi Yi Jie He Za Zhi 2014;34:575-80.

Mailankot M, Kunnath AP, Jayalekshmi H, et al. Radio frequency electromagnetic radiation (RF-EMR) from GSM (0.9/1.8GHz) mobile phones induces oxidative stress and reduces sperm motility in rats. Clinics 2009;64:561-5.

Manta AK, Stravopodis DJ, Papassideri IS, et al. Reactive oxygen species elevation and recovery in Drosophila bodies and ovaries following short-term and long-term exposure to DECT base EMF. Electromagn Biol Med 2014;33:118-31.

Marjanovic AM, Pavicic I, Trosic I. Cell oxidation–reduction imbalance after modulated radiofrequency radiation. Electromagn Biol Med. 2015;34:381-6.

Marjanovic Cermak AM, Pavicic I, Trosic I. Oxidative stress response in SH-SY5Y cells exposed to short-term 1800 MHz radiofrequency radiation. J Environ Sci Health Part A 2018;53:132-8.

Marzook EA, Abd El Moneim, AE, Elhadary AA. Protective role of sesame oil against mobile base station-induced oxidative stress. J Radiat Res Appl Sci. 2014;7:1-6.

Masoumi A, Karbalaei N, Mortazavi S, et al. Radiofrequency radiation emitted from Wi-Fi (2.4 GHz) causes impaired insulin secretion and increased oxidative stress in rat pancreatic islets. Int J Radiat Biol. 2018;94:850-7.

Meena R, Kumari K, Kumar J, et al. Therapeutic approaches of melatonin in microwave radiations-induced oxidative stress-mediated toxicity on male fertility pattern of Wistar rats. Electromagn Biol Med. 2013;33:81-91.

Megha K, Deshmukh PS, Banerjee BD, et al. Microwave radiation induced oxidative stress, cognitive impairment and inflammation in brain of Fischer rats. Indian J Exp Biol. 2012;50:889-96.

Meral I, Mert H, Mert N, et al. Effects of 900-MHz electromagnetic field emitted from cellular phone on brain oxidative stress and some vitamin levels of guinea pigs. Brain Res. 2007;1169:120-4.

Motawi T, Darwish H, Moustafa Y, et al. Biochemical modifications and neuronal damage in brain of young and adult rats after long-term exposure to mobile phone radiations. Cell Biochem Biophys. 2014;70:845-55.

Moustafa YM, Moustafa RM, Belacy A, et al. Effects of acute exposure to the radiofrequency fields of cellular phones on plasma lipid peroxide and antioxidase activities in human erythrocytes. J Pharm Biomed Anal. 2001;26:605-8.

Naziroglu M, Celik O, Ozgul C, et al. Melatonin modulates wireless (2.45 GHz)-induced oxidative injury through TRPM2 and voltage gated Ca(2+) channels in brain and dorsal root ganglion in rat. Physiol Behav. 2012;105:683-92.

Naziroglu M, Cig B, Dogan S, et al. 2.45-Gz wireless devices induce oxidative stress and proliferation through cytosolic Ca(2)(+) influx in human leukemia cancer cells. Int J Radiat Biol. 2012;88:449-56.

Naziroglu M, Gumral N. Modulator effects of L-carnitine and selenium on wireless devices (2.45 GHz)-induced oxidative stress and electroencephalography records in brain of rat. Int J Radiat Biol. 2009;85:680-9.

Ni S, Yu Y, Zhang Y, et al. Study of oxidative stress in human lens epithelial cells exposed to 1.8 GHz radiofrequency fields. PLoS One. 2013; 8:e72370. doi: 10.1371/journal.pone.0072370.

Oksay T, Naziroğlu, M, Doğan, S, et al. Protective effects of melatonin against oxidative injury in rat testis induced by wireless (2.45 GHz) devices. Andrologia 2014;46:65-72.

Oktem F, Ozguner, F, Mollaoglu, H, et al. Oxidative damage in the kidney induced by 900-MHz-emitted mobile phone: protection by melatonin. Arch Med Res. 2005;36:350-5.

Oral B, Guney M, Ozguner F, et al. Endometrial apoptosis induced by a 900-MHz mobile phone: preventive effects of vitamins E and C. Adv Ther. 2006;23:957-73.

Oyewopo A, Olaniyi S, Oyewopo C, et al. Radiofrequency electromagnetic radiation from cell phone causes defective testicular function in male Wistar rats. Andrologia 2017;49:e12772. doi: 10.1111/and.12772.

Ozguner F, Altinbas A, Ozaydin M, et al. Mobile phone-induced myocardial oxidative stress: protection by a novel antioxidant agent caffeic acid phenethyl ester. Toxicol Ind Health. 2005;21:223-30.

Ozguner F, Bardak Y, Comlekci, S. Protective effects of melatonin and caffeic acid phenethyl ester against retinal oxidative stress in long-term use of mobile phone: a comparative study. Mol Cell Biochem. 2006;282:83-8.

Ozguner F, Oktem F, Ayata A, et al. A novel antioxidant agent caffeic acid phenethyl ester prevents long-term mobile phone exposure-induced renal impairment in rat. Prognostic value of malondialdehyde, N-acetyl-beta-D-glucosaminidase and nitric oxide determination. *Mol* Cell Biochem. 2005;277:73-80.

Ozgur E, Guler G, Seyhan N. Mobile phone radiation-induced free radical damage in the liver is inhibited by the antioxidants N-acetyl cysteine and epigallocatechin-gallate. Int J Radiat Biol. 2010;86:935-45.

Ozgur E, Kismali G, Guler, G, et al. Effects of prenatal and postnatal exposure to GSM-like radiofrequency on blood chemistry and oxidative stress in infant rabbits, an experimental study. Cell Biochem Biophys. 2013;67:743-51.

Özorak A, Nazıroğlu M, Çelik Ö, et al. Wi-Fi (2.45 GHz)- and mobile phone (900 and 1800 MHz)-induced risks on oxidative stress and elements in kidney and testis of rats during pregnancy and the development of offspring. Biol Trace Elem Res. 2013;156:221-9.

Özsobacı NP, Ergün D D, Durmuş S, et al. Selenium supplementation ameliorates electromagnetic field-induced oxidative stress in the HEK293 cells. J Trace Elem Med Biol. 2018;50:572-9.

Özsobacı NP, Ergün DD, Tunçdemir M, et al. Protective effects of zinc on 2.45 ghz electromagnetic radiation-induced oxidative stress and apoptosis in HEK293 cells. Biol Trace Elem Res.2020;194:368-78.

Pandey N, Giri S. Melatonin attenuates radiofrequency radiation (900 MHz)-induced oxidative stress, DNA damage and cell cycle arrest in germ cells of male Swiss albino mice. Toxicol Ind Health. 2018;34:315-27.

Pilla AA. Electromagnetic fields instantaneously modulate nitric oxide signaling in challenged biological systems. Biochem Biophys Res Commun. 2012;426:330-3.

Qin F, Yuan H, Nie J, et al. Effects of nano-selenium on cognition performance of mice exposed in 1800 MHz radiofrequency fields]. Wei Sheng Yan Jiu. 2014;43:16-21.

Ragy MM. Effect of exposure and withdrawal of 900-MHz-electromagnetic waves on brain, kidney and liver oxidative stress and some biochemical parameters in male rats. Electromagn Biol Med. 2015;34:279-84.

Saikhedkar N, Bhatnagar M, Jain A, et al. Effects of mobile phone radiation (900 MHz radiofrequency) on structure and functions of rat brain. Neurol Res. 2014;36:1072-9.

Saygin M, Ozmen O, Erol O, et al. The impact of electromagnetic radiation (2.45 GHz, Wi-Fi) on the female reproductive system: The role of vitamin C. Toxicol Ind Health. 2018;34:620-30.

Sefidbakht Y, Moosavi-Movahedi AA, Hosseinkhani S, et al. Effects of 940 MHz EMF on bioluminescence and oxidative response of stable luciferase producing HEK cells. Photochem Photobiol Sci. 2014;13:1082-92.

Shaheen W, Amer NM, Hafez SF, et al. Effect of antioxidants intake on oxidative stress among mobile phone users. Egypt J Chem. 2021;64:3903-12.

Shahin S, Singh VP, Shukla RK, et al. 2.45 GHz microwave irradiation-induced oxidative stress affects implantation or pregnancy in mice, Mus musculus. Appl Biochem Biotechnol. 2013;169:1727-51.

Sharma A, Shrivastava S, Shukla S. Exposure of radiofrequency electromagnetic radiation on biochemical and pathological alterations. Neurol India. 2020;68:1092-1100.

Sharma S, Shukla S. Effect of electromagnetic radiation on redox status, acetylcholine esterase activity and cellular damage contributing to the diminution of the brain working memory in rats. J Chemical Neuroanat. 2020;106:101784. doi: 10.1016/j.jchemneu.2020.101784.

Sharma VP, Singh HP, Kohli RK, et al. Mobile phone radiation inhibits Vigna radiata (mung bean) root growth by inducing oxidative stress. Sci Total Environ. 2009;407:5543-7.

Shedid SM, El-Tawill GA, Algeda FR, et al. The impact of 950MHz electromagnetic radiation on the brain and liver of rats and the role of garlic treatment. Egypt J Rad Sci Applic. 2019;32:51-60.

Singh HP, Sharma VP, Batish DR, et al. Cell phone electromagnetic field radiations affect rhizogenesis through impairment of biochemical processes. Environ Monit Assess. 2012;184:1813-21.

Sokolovic D, Djindjic B, Nikolic J, et al. Melatonin reduces oxidative stress induced by chronic exposure of microwave radiation from mobile phones in rat brain. J Radiat Res. 2008;49:579-86.

Sokolovic D, Djordjevic B, Kocic G, et al. Melatonin protects rat thymus against oxidative stress caused by exposure to microwaves and modulates proliferation/apoptosis of thymocytes. Gen Physiol Biophys. 2013;32:79-90.

Tkalec M, Malaric K, Pevalek-Kozlina B. Exposure to radiofrequency radiation induces oxidative stress in duckweed Lemna minor L. Sci Total Environ. 2007;388:78-89.

Tkalec M, Stambuk A, Srut M, et al. Oxidative and genotoxic effects of 900 MHz electromagnetic fields in the earthworm Eisenia fetida.. Ecotoxicol Environ Saf. 2013;90:7-12.

Tök L, Nazıroğlu M, Doğan S, et al. Effects of melatonin on Wi-Fi-induced oxidative stress in lens of rats. Indian JOphthalmol. 2014;62:12-15.

Tomruk A, Guler G, Dincel AS. The influence of 1800 MHz GSM-like signals on hepatic oxidative DNA and lipid damage in nonpregnant, pregnant, and newly born rabbits. Cell Biochem Biophys. 2010;56:39-47.

Topsakal S, Ozmen O, Cicek E, et al. The ameliorative effect of gallic acid on pancreas lesions induced by 2.45 GHz electromagnetic radiation (Wi-Fi) in young rats. J Radiat Res Appl Sci. 2017;10:233-40.

Tsybulin O, Sidorik E, Kyrylenko S, et al. GSM 900 MHz microwave radiation affects embryo development of Japanese quails. Electromagn Biol Med. 2012;31:75-86.

Türedi S, Hancı H, Topal Z, et al. The effects of prenatal exposure to a 900-MHz electromagnetic field on the 21-day-old male rat heart. Electromagn Biol Med. 2015;34:390-7.

Turker Y, Naziroglu M, Gumral N, et al. Selenium and L-carnitine reduce oxidative stress in the heart of rat induced by 2.45-GHz radiation from wireless devices. Biol Trace Elem Res. 2011;143:1640-50.

Usman JD, Isyaku UM, Magaji RA, et al. Assessment of electromagnetic fields, vibration and sound exposure effects from multiple transceiver mobile phones on oxidative stress levels in serum, brain and heart tissue. Sci Afr.. 2020;7:e00271. <https://doi.org/10.1016/j.sciaf.2020.e00271>

Wang M, Yang G, Li Y, et al. Protective role of Vitamin C in Wi-Fi induced oxidative stress in MC3T3-E1 cells in vitro. Appl Comput Electromagn Soc J. 2020;35:online.

Xu S, Zhou Z, Zhang L, et al. Exposure to 1800 MHz radiofrequency radiation induces oxidative damage to mitochondrial DNA in primary cultured neurons. Brain Res. 2010;1311:189-96.

Yakymenko I, Burlaka A, Tsybulin A, et al. Oxidative and mutagenic effects of low intensity GSM 1800 MHz microwave radiation. Exp Oncol. 2018;40:282-7.

Yurekli AI, Ozkan M, Kalkan T, et al. GSM base station electromagnetic radiation and oxidative stress in rats. Electromagn Biol Med. 2006;25:177-88.

Zmyślony M, Politanski P, Rajkowska E, et al. Acute exposure to 930 MHz CW electromagnetic radiation in vitro affects reactive oxygen species level in rat lymphocytes treated by iron ions. Bioelectromagnetics. 2004;25:324-8.

Zosangzuali M, Lalremruati, M, Lalmuansangi C, et al. Effects of radiofrequency electromagnetic radiation emitted from a mobile phone base station on the redox homeostasis in different organs of Swiss albino mice. Electromagn Biol Med. 2021;40:393-407.

**Table 2. Studies that reported no significant oxidative effects of RFR exposure**

| **Reference** | **Biological system exposed** | **RFR exposure** | **Effects reported** |
| --- | --- | --- | --- |
| ([Hook et al., 2004](#_ENREF_6)) | Mammalian cells *in vitro* | 835.62 MHz (frequency-modulated continuous-wave, FMCW) and 847.74 MHz (code division multiple access, CDMA), SAR=0.8 W/kg, for 20–22 h | FMCW- and CDMA-modulated RFR did not alter parameters indicative of oxidative stress. |
| ([Ferreira, Bonatto, et al., 2006](#_ENREF_4)) | Rat whole body | 800-1800 MHz, from cell phone | No changes in lipid and protein damage, and in non-enzymatic antioxidant defense in frontal cortex or hippocampus. |
| ([Ferreira, Knakievicz, et al., 2006](#_ENREF_5)) | Pregnant rat whole body | RFR from cell phone | No differences in oxidative parameter of offspring blood and liver, but increase in erythrocytes micronuclei incidence in offspring. |
| ([Dasdag et al., 2003](#_ENREF_1)) | Rat whole body | Cell phone RFR, SAR=0.52 W/kg, 20 min/day for 1 month | No alteration in MDA concentration. |
| ([Demirel et al., 2012](#_ENREF_3)) | Rat whole body | 3G cell phone RFR, “standardized daily dose” for 20 days | No difference in GSH-Px and CAT activity in eye tissues, in MDA and GSH levels in blood. |
| ([Khalil et al., 2013](#_ENREF_7)) | Human head/whole body | Cell phone RFR (talking mode) for 15 or 30 min | No relationship between exposure and changes in the salivary oxidant/antioxidant profile. |
| ([de Souza et al., 2014](#_ENREF_2)) | Human head/whole body | Cell phone RFR | No difference in the saliva from the parotid gland exposed to cell phone RFR to the saliva from the opposite gland of each individual. |

Dasdag S, Akdag MZ, Aksen F, et al. Whole body exposure of rats to microwaves emitted from a cell phone does not affect the testes. Bioelectromagnetics. 2003;24:182-188.

de Souza, F. T., Silva, J. F., Ferreira, E. F., et al. (2014). Cell phone use and parotid salivary gland alterations: no molecular evidence. Cancer Epidemiol Biomarkers Prev. 20114;23:1428-31.

Demirel S, Doganay S, Turkoz Y, et al. Effects of third generation mobile phone-emitted electromagnetic radiation on oxidative stress parameters in eye tissue and blood of rats. Cutan Ocul Toxicol. 2012;31:89-94.

Ferreira AR, Bonatto F. de Bittencourt Pasquali MA, et al. Oxidative stress effects on the central nervous system of rats after acute exposure to ultra high frequency electromagnetic fields. Bioelectromagnetics. 2006;27:487-493.

Ferreira AR, Knakievicz T, Pasquali MA, et al. Ultra high frequency-electromagnetic field irradiation during pregnancy leads to an increase in erythrocytes micronuclei incidence in rat offspring. Life Sci. 2006;80:43-50.

Hook GJ, Spitz DR, Sim JE, et al. Evaluation of parameters of oxidative stress after in vitro exposure to FMCW- and CDMA-modulated radiofrequency radiation fields. Radiat Res. 2004;162:497-504.

Khalil AM, Abu Khadra KM, Aljaberi AM, et al. Assessment of oxidant/antioxidant status in saliva of cell phone users. Electromagn Biol Med. 2014;33;92-7.
