# Supplementary material for: Scientific evidence invalidates health assumptions underlying the FCC and ICNIRP exposure limit determinations for radiofrequency radiation: implications for 5G
Source: Environ Health. 2022 Oct 18;21:92. doi: 10.1186/s12940-022-00900-9 (PMC9576312; doi:10.1186/s12940-022-00900-9)
Supplement: Supplementary file 2 — Additional file 2: Appendix 2. On the Inadequacy of the psSAR Dosimetric Parameter at Frequencies above 1 GHz. Table 1. Electric permittivity and electric conductivity of the gray matter. Figure 1. A block of gray matter radiated by different frequencies. The highlighted cubes are of 10 g, 1 g, 100 mg and 10 mg. Fig. 2. A block of gray matter radiated by different frequencies. The highlighted cubes are of 10 g, 1 g, 100 mg and 10 mg. Fig. 3. Electric field intensity averaged in each cube for different frequencies: in the left axis, the electric field is in dB and in the right axis the electric field is in V/m normalized to 100 V/m. [file 12940_2022_900_MOESM2_ESM.docx]

**Appendix 2**

On the Inadequacy of the psSAR Dosimetric Parameter

at Frequencies above 1 GHz

The following example is intended to illustrate the meaning and the limitations when using psSAR as a dosimetric parameter at different frequencies.

The psSAR is calculated by averaging in cubic volumes containing a given amount of mass. The material in the cube is considered to be a homogeneous with a given mass density. This means that the psSAR is calculated in a fixed volume, and implies using fixed-sided cubes measured in fractions of meters.

The previous ICNIRP recommendation limited the use of psSAR averaged in cubic volumes containing 10 grams of tissue (10g-psSAR) to frequencies above 10 GHz. In the recent 2020 review [1] this limitation was reduced to 6 GHz. The FCC recommendation [2-3] is to average in 1 gram (1g-psSAR). Considering densities close to 1 kg per liter, 10 g corresponds to cubes with 2.15 cm side and 1 g to cubes with 1 cm side. These dimensions are used for any frequency, instead of using a parameter (a cube) related to the wavelength, as usual in electrical engineering.

The examples provided below show the limitation of these dosimetric parameters even for frequencies lower than 6 GHz, which have been in use for a long time, and the total inadequacy for the 26 GHz frequency band which should be deployed and in use in the next 5 years in many countries.

The incidence of different frequency plane waves on a block of a homogeneous material with the same electromagnetic parameters values of the gray matter (in Table I), according to the Cole-Cole curve fitting and the measurements performed by C. Gabriel and S. Gabriel [4] were simulated using CST Studio [5].

Table 1: Electric permittivity and electric conductivity of the gray matter

| Frequency in MHz | Permittivity (εr) | Conductivity (σ) in S/m | Skin depth (δ) in mm |
| --- | --- | --- | --- |
| 100 | 80.14 | 0.55946 | 96.94 |
| 900 | 52.725 | 0.94227 | 41.54 |
| 1,800 | 50.079 | 1.3913 | 27.26 |
| 2,450 | 48.911 | 1.8077 | 20.72 |
| 3,500 | 47.305 | 2.636 | 13.99 |
| 5,800 | 44.004 | 4.9865 | 7.17 |
| 10,000 | 38.112 | 10.31 | 3.27 |
| 18,000 | 28.704 | 20.496 | 1.46 |
| 26,000 | 22.242 | 28.551 | 0.95 |

The simulated frequencies are 100 MHz (broadcasting), 900 MHz (first cell phone generation), 1800 MHz (second cell phone generation), 2450 MHz (WiFi), 3500 MHz (5G), 5800 MHz (WiFi), 10 GHz, 18 GHz and 26 GHz (5G) and the cubes depicted are of 2.15 cm side (for 10 g), 1 cm side (1 g), 4.6 mm side (100 mg) and 2.15 mm side (10 mg). Care has been taken in order to avoid mathematical artifacts in the FDTD method which could lead to non-realistic results. Also, convergence and instabilities where considered.

Figure 1 and Figure 2 shows the electric field intensity in V/m on a 50 dB scale for the nine selected frequencies. Each color tone represents a 2 dB range.


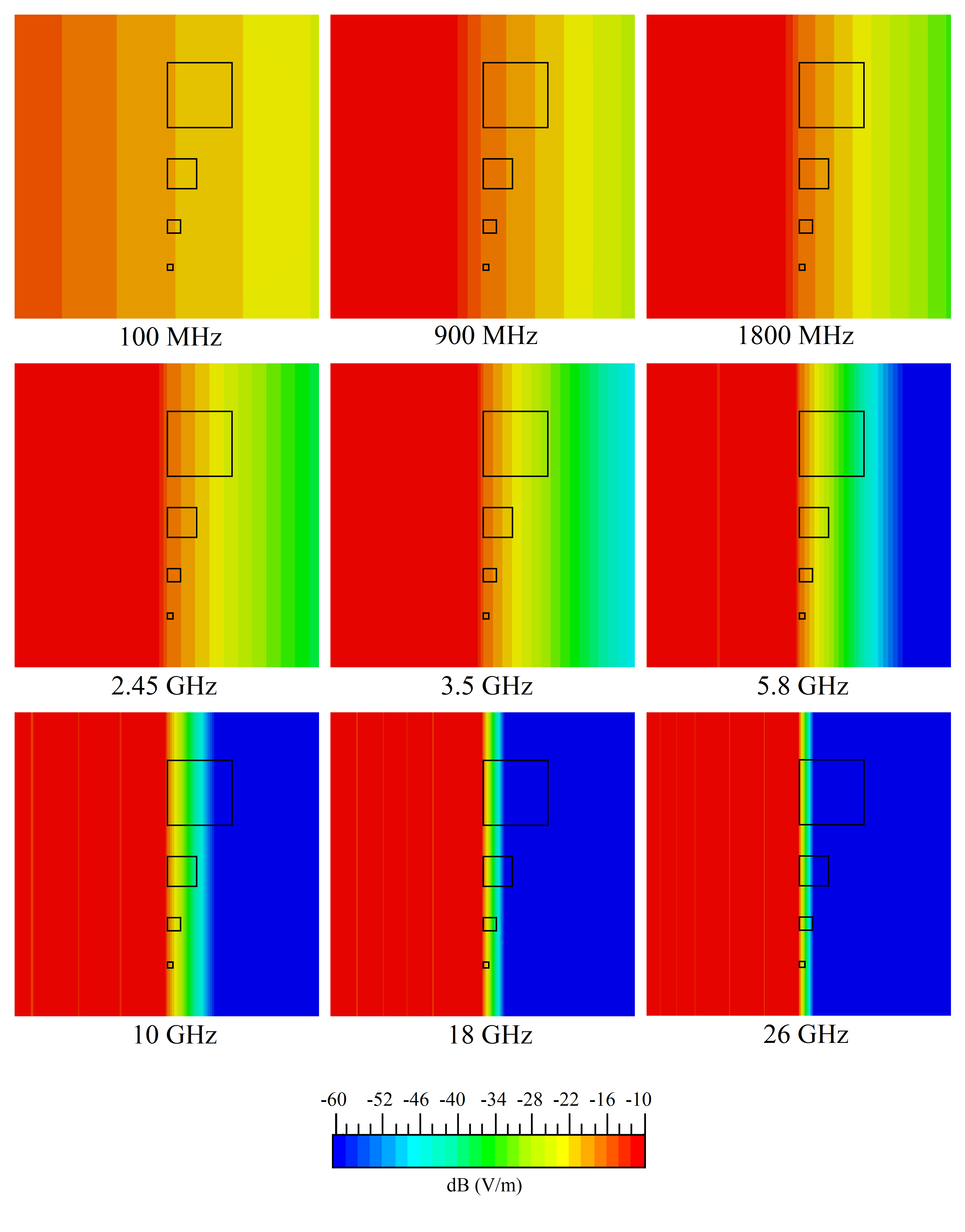
Fig 1: A block of gray matter radiated by different frequencies. The highlighted cubes are of 10 g, 1 g, 100 mg and 10 mg.

**
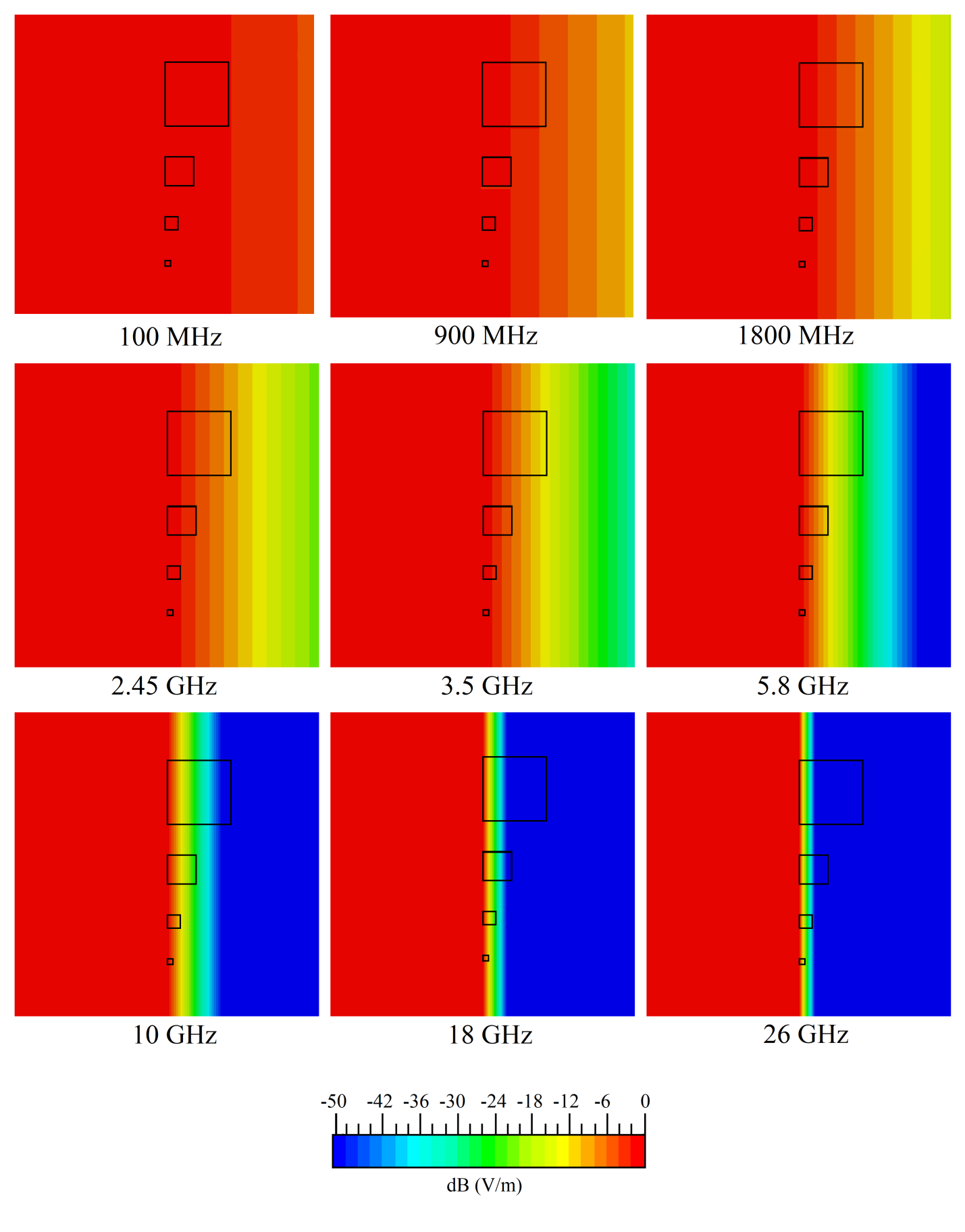
**

Fig 2: A block of gray matter radiated by different frequencies. The highlighted cubes are of 10 g, 1 g, 100 mg and 10 mg.

It can be seen that for the smaller cubes and lower frequencies averaging in the cube does not underestimate the maximum value on the cube surface, but for higher frequencies the psSAR averaged on larger cubes can be several dB lower than the psSAR averaged on smaller cubes, allowing higher electric field intensities and EMF absorption in the surface. In Figure 1 the scale is from -10 dB to -50 dB while in Figure 2 the scale maximum is the electric field intensity at the surface of the block.

These results show that, for 2.45 GHz, averaging over 10 g underestimates by 4 dB the psSAR averaged in smaller cubes (more than two times underestimation) and for 5.8 GHz, averaging over a 10 g cube underestimates psSAR by 12 dB (almost 20 times underestimation) compared with averaging in a 10 mg cube and by 6 dB (four times underestimation) with averaging on a 1 g cube. Further examples will be included with a four-layer flat phantom (considering the skin, muscle, skull and gray matter) in which similar results will be shown.


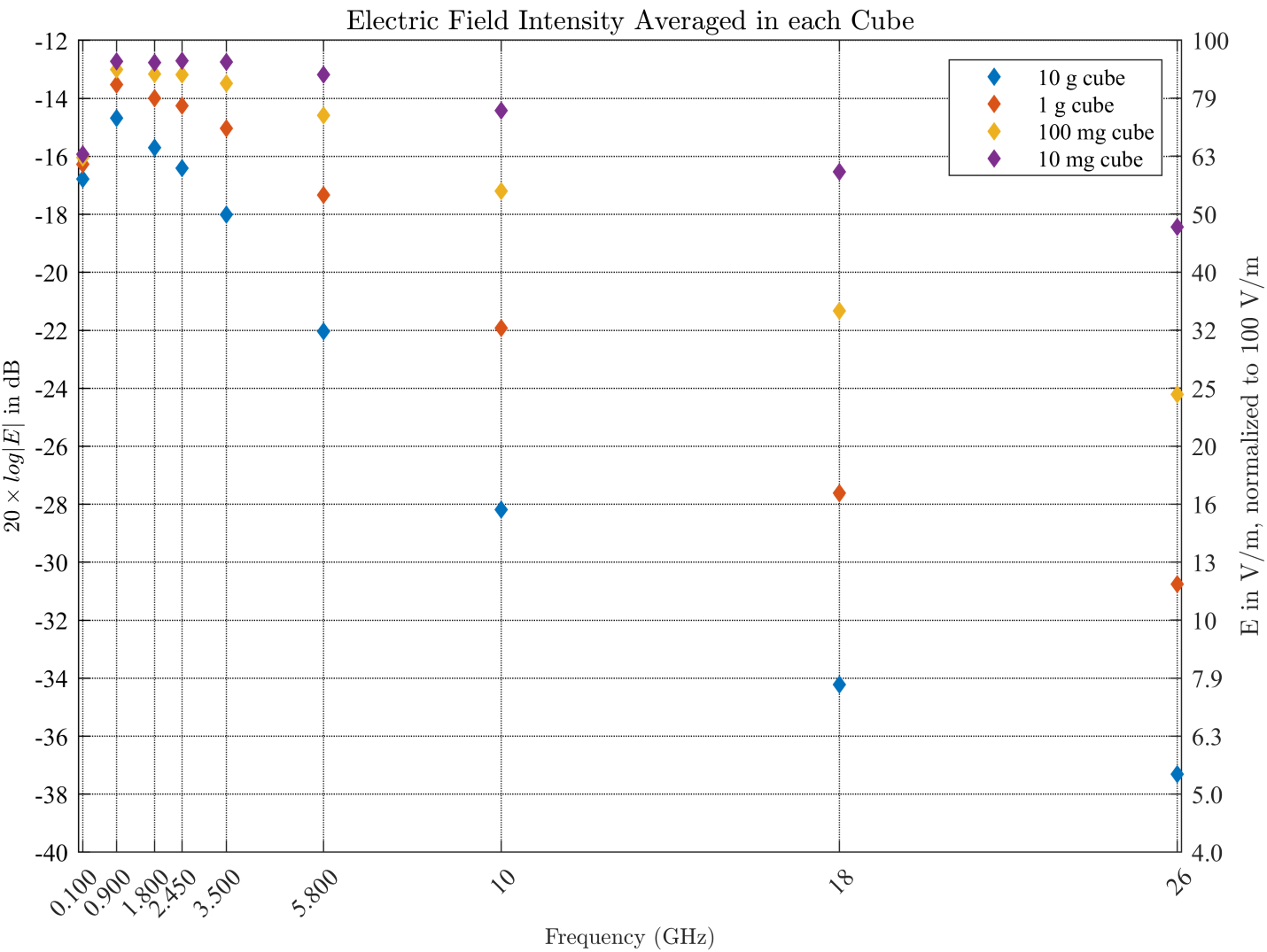


Fig 3 Electric field intensity averaged in each cube for different frequencies: in the left axis, the electric field is in dB and in the right axis the electric field is in V/m normalized to 100 V/m

In Fig. 3, it is shown that when the frequency is increased, the difference (or underestimation) between the E field (and the psSAR) averaged in larger cubes (e.g. 10 g or 1 g) compared to smaller cubes (e.g. 100 mg and 10 mg) becomes more pronounced. For example, at 5.8 GHz the difference between the psSAR averaged in a 10 g cube and in a 10 mg cube is around 9 dB (which is an almost ten times underestimation). Also, considering the 10 g cube, the difference between the psSAR for a 5.8 GHz EMF compared to a 0.9 GHz EMF is around 7 dB (or more than four times underestimation)

The analyses shown above demonstrate that ICNIRP’s 10g-psSAR [1] and FCC’s 1g-psSAR [2-3] recommendations do not provide realistic dosimetric parameters to evaluate and limit EMF exposures and absorption above 1 GHz, and therefore, the recommended cube volumes should be substantially reduced.

**References**

[1] International Commission on Non-Ionizing Radiation Protection (ICNIRP). Guidelines for Limiting Exposure to Electromagnetic Fields (100 kHz to 300 GHz), Health Physics 2020;118:483–524.

[2] Federal Communications Commission (FCC). 1997. Evaluating Compliance with FCC Guidelines for Human Exposure to Radiofrequency Electromagnetic Fields, OET Bulletin 65. https://transition.fcc.gov/Bureaus/Engineering_Technology/Documents/bulletins/oet65/oet65.pdf

[3] Federal Communications Commission (FCC). 2019. Proposed Changes in the Commission's Rules Regarding Human Exposure to Radiofrequency Electromagnetic Fields; Reassessment of Federal Communications Commission Radiofrequency Exposure Limits and Policies. FCC19-126. <https://www.federalregister.gov/documents/2020/04/06/2020-06966/human-exposure-to-radiofrequency-electromagnetic-fields>

[4] Gabriel C, S. Gabriel S. 1996. ‘Compilation of the dielectric properties of body tissues at RF and microwaves frequencies. Tech. Rep. AL/OE-TR-1996-0037, Jun. 1996. [Online]. Available: <http://niremf.ifac.cnr.it/docs/DIELECTRIC/Report.html>

[5] Dassault Systèmes, CST Studio”. Available: <https://www.3ds.com/products-services/simulia/products/cst-studio-suite/>
